# Dynamics of self-sustained asynchronous-irregular activity in random networks of spiking neurons with strong synapses

**DOI:** 10.3389/fncom.2014.00136

**Published:** 2014-10-30

**Authors:** Birgit Kriener, Håkon Enger, Tom Tetzlaff, Hans E. Plesser, Marc-Oliver Gewaltig, Gaute T. Einevoll

**Affiliations:** ^1^Neural Coding and Dynamics, Center for Learning and Memory, University of Texas at AustinAustin, TX, USA; ^2^Computational Neuroscience, Department of Mathematical Sciences and Technology, Norwegian University of Life SciencesÅs, Norway; ^3^Simula Research Laboratory, Kalkulo ASFornebu, Norway; ^4^Institute of Neuroscience and Medicine (INM-6), Computational and Systems Neuroscience and Institute for Advanced Simulation (IAS-6), Theoretical Neuroscience, Jülich Research Centre and JARAJülich, Germany; ^5^Blue Brain Project, In-Silico Neuroscience - Cognitive Architectures, École Polytechnique Fédérale de LausanneLausanne, Switzerland; ^6^Department of Physics, University of OsloOslo, Norway

**Keywords:** Self-sustained activity, leaky integrate-and-fire neurons, balanced random networks, linear stability, spike train irregularity

## Abstract

Random networks of integrate-and-fire neurons with strong current-based synapses can, unlike previously believed, assume stable states of sustained asynchronous and irregular firing, even without external random background or pacemaker neurons. We analyze the mechanisms underlying the emergence, lifetime and irregularity of such self-sustained activity states. We first demonstrate how the competition between the mean and the variance of the synaptic input leads to a non-monotonic firing-rate transfer in the network. Thus, by increasing the synaptic coupling strength, the system can become bistable: In addition to the quiescent state, a second stable fixed-point at moderate firing rates can emerge by a saddle-node bifurcation. Inherently generated fluctuations of the population firing rate around this non-trivial fixed-point can trigger transitions into the quiescent state. Hence, the trade-off between the magnitude of the population-rate fluctuations and the size of the basin of attraction of the non-trivial rate fixed-point determines the onset and the lifetime of self-sustained activity states. During self-sustained activity, individual neuronal activity is moreover highly irregular, switching between long periods of low firing rate to short burst-like states. We show that this is an effect of the strong synaptic weights and the finite time constant of synaptic and neuronal integration, and can actually serve to stabilize the self-sustained state.

## 1. Introduction

The sustained activity of populations of spiking neurons, even in the absence of external input, is observed in many circumstances, amongst them spontaneously active neurons in cell cultures (see e.g., Marom and Shahaf, 2002; Wagenaar et al., 2006), *in vitro* slice preparations (see e.g., Plenz and Aertsen, 1996; Mao et al., 2001; Cossart et al., 2003; Shu et al., 2003) and even *in toto* preparations of whole brain parts, such as cortical slabs (Burns and Webb, 1979; Timofeev et al., 2000) or the entire hippocampus (Ikegaya et al., 2013). Another prominent phenomenon in this context is the existence of up and down states in striatum and cortex, i.e., states in which neurons switch between two preferred membrane potentials: In the so-called down-state membrane potentials are close to the resting value, corresponding to a quiescent state, while in the so-called up-states membrane potentials are at a depolarized level that allows for the emission of spikes. These states are observed both *in vivo* (Steriade et al., 1993, 2001) and *in vitro* (Sanchez-Vives and McCormick, 2000; Cossart et al., 2003; Shu et al., 2003). Finally, the persistent activation of groups of neurons is a key element of working memory, the so-called *delay activity*, which is commonly observed in the prefrontal cortex of awake behaving monkeys during active memory tasks, where animals have to remember a presented stimulus after it is removed (Goldman-Rakic, 1995).

Several possible explanations of how neuronal networks can generate and sustain activation of subpopulations of neurons have been put forward in the past, amongst them persistent activation by thalamo-cortical and cortico-cortical loops, intrinsic cellular bistability, or attractor states of local recurrent networks (Wang, 2001; Compte, 2006). Especially the latter idea inspired a lot of research in the framework of spiking neuronal networks (e.g., Compte et al., 2000, 2003a,b; Brunel, 2003; Vogels and Abbott, 2005; Compte, 2006; Holcman and Tsodyks, 2006; Renart et al., 2007; Kumar et al., 2008; Destexhe, 2009) and neural-field models (e.g., Wilson and Cowan, 1972, 1973; Amari, 1977; Laing and Chow, 2001; Coombes, 2005). One important element required for stable persistent activation in network models is strong excitatory feedback, while inhibition is needed to keep the system from entering a state of run-away excitation.

Of particular interest is the question of what constitutes the *minimal* cortical architecture to generate sustained activity states, especially states that stay active even without additional external or non-local input.

Griffith (1963) already presented a general proof of principle that abstract networks of excitatory and inhibitory neurons can stably sustain states of persistent ongoing activity. Kumar et al. (2008), Vogels and Abbott (2005), and El Boustani and Destexhe (2009) showed, moreover, how balanced random networks of leaky integrate-and-fire (LIF) neurons with conductance-based synapses can sustain states of elevated rate in the absence of external input. This is due to a non-monotonic input-output firing-rate function resulting from the shunting of membrane-potential fluctuations and a modulation of the effective membrane time constants (Kuhn et al., 2004).

In most of these models, attractor states are characterized by rather constant individual firing rates and homogeneous population activity. In experimental investigations of sustained states in prefrontal cortex during working memory (Marder et al., 1996; Wang, 2001; Compte et al., 2003a; Compte, 2006; Druckmann and Chklovskii, 2012) and also up-states (Shu et al., 2003), however, it is observed that individual neurons vary a lot in their relative contribution to the local population activity over time, with periods of both silence and elevated rates, while the compound activity persists. A computational model of self-sustained activity should reproduce this pronounced irregularity in the spike times of individual neurons.

Here, we demonstrate that LIF neurons with current-based synapses can sustain highly irregular activity at moderate rates provided the coupling between them is sufficiently strong (see also the preprint by Gewaltig, 2013). That strong weights indeed occur in neuronal networks was demonstrated in thorough experimental investigations that showed that distributions of synapse strength *J* in cortex are log-normally distributed, with many weak and some very strong synapses leading to postsynaptic-potential (PSP) peak-amplitudes of up to a few millivolts (see e.g., Song et al., 2005; Lefort et al., 2009; Avermann et al., 2012). The same was observed for inter-pyramidal synapses in hippocampus (Ikegaya et al., 2013). These few but strong synapses suffice to allow self-sustained asynchronous-irregular (SSAI) activity, provided the relative inhibitory strength *g* is in the right range. Teramae et al. (2012) and Ikegaya et al. (2013) have studied similar effects in networks of neurons with conductance-based synapses. Here, we show by numerical simulations that there is a distinct transition in the *g*-*J*-plane above which the system jumps to very large, virtually infinite lifetimes of persistent activity, and thus appears to become stable.

We demonstrate by simple arguments how the competition between the mean and variance of the neuronal input as a function of synaptic strength leads to a non-monotonic firing-rate transfer in the network. Thus, by increasing the synaptic coupling strength the system can become bistable, and in addition to the quiescent state a second stable fixed point at moderate firing rates, the SSAI state, can emerge by a saddle-node bifurcation. The population activity in this SSAI state is characterized by inherent population fluctuations and highly irregular spiking of individual neurons.

We show that the high irregularity in the activity of individual cells is induced by the large fluctuations of the neuronal input currents which keep the membrane potential far away from threshold for long times and induce firing at close to maximal rate when there is a large occasional suprathreshold transient. Hence, the firing-rate activity of individual neurons is basically binary. In particular, it demonstrates that highly irregular individual neuron firing and stable sustained activity states are indeed compatible and do not necessitate extra sources of variability, such as additional external noise or cellular bistability.

The substantial population fluctuations on the other hand lead to a constant perturbation of the network activity from the SSAI-attractor. We show how taking this into account in a simple escape rate model can explain the observed lifetimes of the persistent activation as a function of the network coupling parameters *g* and *J*: If the fluctuations are too strong, the system can escape the basin of attraction and activity spontaneously breaks down, while for other *g*-*J*-pairs the escape probability becomes very small and the system is virtually stable on biologically relevant time scales.

The paper is organized as follows: In Section 2 we will shortly outline the neuron and network model as well as the data analysis techniques used in this paper. In Section 3.1 we present the essential features of the SSAI-state in strongly coupled networks, and then explain the mechanism underlying its emergence and irregularity in Section 3.2. Section 3.3 discusses the effect of synaptic weight distributions on the emergence of SSAI. In Section 3.4 we show how a stochastic rate model can capture the distribution of lifetimes observed in simulations, and in Section 4 we finally summarize and discuss our results.

## 2. Materials and methods

### 2.1. Network model

We study balanced random networks (van Vreeswijk and Sompolinsky, 1996; Brunel, 2000) of *N* leaky integrate-and-fire (LIF) neurons with current-based synapses. Each network is composed of *N_E_* excitatory and *N_I_* = γ*N_E_* inhibitory neurons. Throughout the article, we assume γ = 1/4; see Tables 1, 2 for a concise summary of models and parameters following Nordlie et al. (2009). The network topology is random, i.e., all neurons are connected independently with equal probability ϵ ∈ [0, 1], irrespective of their identity.

**Table 1:** **Model and simulation description**.

**A**	**MODEL SUMMARY**	
Populations	Three: excitatory (E), inhibitory (I), external input (*E*_ext_)	
Connectivity	Random convergent connectivity with probability ϵ	
Neuron model	Leaky integrate-and-fire (LIF), fixed voltage threshold, exact integration scheme (Rotter and Diesmann, 1999) (update every 0.1 ms)	
Synapse model	α-shaped post-synaptic current (PSC)	
Input	Independent Poisson spike trains	
**B**	**POPULATIONS**	
**Name**	**Elements**	**Size**
E,I	LIF neuron	*N_E_*, *N_I_* = γ*N_E_*
E_ext_	Poisson generator	*N*_ext_ = *N_E_* + *N_I_*
**C**	**CONNECTIVITY**	
**Source**	**Target**	**Pattern**
{E,I}	E ∪ I	Random convergent *C_E_* = ϵ*N_E_* → 1, *C_I_* = ϵ*N_I_* → 1
E_ext_	E ∪ I	Non-overlapping 1→ 1
**D**	**NEURON AND SYNAPSE MODEL**	
Name	Leaky integrate-and-fire neuron with α-shaped PSCs	
Subthreshold dynamics	$\begin{array}{l} \tau_{\text{m}}{\dot{V}}_{i}(t)=-V_{i}(t)+R_{\text{m}}\left(I_{\text{syn},i}(t-d)+I_{\text{ext},i}(t)\right)\text{ if }t>t^{*}+\tau_{\text{ref}}\\ \;V_{i}(t)\;=V_{\text{res}}\text{ else} \end{array}$	
Spiking	If *V*(*t* −) < *V*_thr_ ∧ *V*(*t* +) ≥ *V*_thr_	
	1. Set spike time t^*^ = *t*	
	2. Emit spike with time-stamp *t_k_* = t^*^	
Postsynaptic currents	$\begin{array}{l} \;I_{\text{syn},i}(t)=\sum_{j,k}{\text{PSC}}_{ij}(t-t_{j,k})\text{ Network input current of neuron }i\\ \;I_{\text{ext},i}(t)=\sum_{k}{\text{PSC}}_{\text{ext},i}(t-t_{k})\text{ External input current of neuron }i\\ \;{\text{PSC}}_{ij}(t)=A(J_{ij})\frac{t}{\tau_{\text{syn}}}e^{1-t/\tau_{\text{syn}}}H(t),\;J_{ij}\in \{-gJ,0,J\}\\ {\text{PSC}}_{\text{ext},i}(t)=A(J)\frac{t}{\tau_{\text{syn}}}e^{1-t/\tau_{\text{syn}}}H(t) \end{array}$	
**E**	**INPUT**	
**Type**	**Description**	
Poisson generators	Spike times *t_k_* in *I*_ext_(*t*) are Poisson point processes of rate ν_ext_	

**Table 2:** **Default parameters**.

**A**	**CONNECTIVITY**	
**Name**	**Value**	**Description**
*N_E_*	{100000, 5000}	Number of excitatory neurons (**Figures 1**–**4**, **10**, **11**, and **Figures 5**–**9**, resp.)
*N_I_*	γ*N_E_*, γ = 1/4	Number of inhibitory neurons
ϵ	{0.01, 0.1}	Connection density (**Figures 1**–**4**, **10**, **11**, and **Figures 5**–**9**, resp.)
**B**	**NEURON**	
**Name**	**Value**	**Description**
τ_m_	20 ms	Membrane time constant
*R*_m_	20 GΩ	Membrane resistance
*V*_thr_	20 mV	Firing threshold
*V*_res_	0 mV	Reset potential
τ_ref_	2 ms	Refractory time
**C**	**SYNAPSES**	
**Name**	**Value**	**Description**
*J*	∈ [0.1, 4.5] mV	Peak-amplitude of excitatory PSP(*t*)
*A*(*J*)	∈ [0.12, 5.44] pA	Amplitude of excitatory PSC(*t*) for α-current input, normalized such that peak-amplitude of PSP(*t*) = *J*
*g*	∈ [4., 8.]	Relative inhibitory coupling strength
*d*	1.5 ms	Synaptic delay
τ_syn_	0.5 ms	synaptic time constant
**D**	**INPUT**	
**Name**	**Value**	**Description**
ν_ext_	1000(*V*_thr_ − *V*_res_)/*e*τ_syn_*R_m_A(J)*	Rate of external Poisson stimulus
*t*_stim_	1000 ms	Stimulus duration

Though all results we present below hold for a very broad class of balanced random networks, all neurons in the simulations presented here received the same number of excitatory and inhibitory synapses, i.e., *C_E_* = ϵ*N_E_* and *C_I_* = ϵ*N_I_*, respectively. Here, we will use ϵ ∈ {0.01, 0.1} which spans connection probabilities observed in local cortical networks.

Finally, we assume that the coupling strength is parametrized by the peak-amplitude *J*_*ij*_ of the postsynaptic potential (PSP) that is evoked in a neuron *i* in response to incoming spikes, such that

(1) $$J_{ij}=\{\begin{array}{ll} J & \text{if the presynaptic neuron }j\text{ is excitatory},\\ -gJ & \text{if the presynaptic neuron }j\text{ is inhibitory},\\ 0 & \text{if the synapse }j\to i\text{ does not exist} \end{array}.$$

We emphasize that the main results do not crucially depend on the network density or the fine details of the weight and degree distribution.

### 2.2. Neuron model

The dynamics of the subthreshold membrane potential *V_i_*(*t*) of neuron *i* is linear and governed by

(2) $$\tau_{\text{m}}{\dot{V}}_{i}(t)=-V_{i}(t)+R_{\text{m}}I_{\text{syn},i}(t-d)+R_{\text{m}}I_{\text{ext},i}\;(t)$$

with membrane time constant τ_*m*_, membrane resistance *R*_m_, a finite transmission delay *d*, the total synaptic input current *I*_syn_,*i* resulting from the local-network activity, and the external current *I*_ext_,*i*(*t*). The synaptic input current is given by the linear superposition of post-synaptic currents, i.e., *I*_syn_,*i* (*t*) = ∑_*j* ∈ Pre[i]_ ∑_*k*_ PSC_*ij*_(*t* − *t_j,k_*), where Pre[*i*] denotes the set of presynaptic neurons of neuron *i*, and *k* denotes the *k*-th spike emission of neuron *j* ∈ Pre[*i*]. The post-synaptic current PSC_*ij*_(*t*) is given by

(3) $${\text{PSC}}_{ij}(t)=A(J_{ij})\frac{t}{\tau_{\text{syn}}}e^{1-t/\tau_{\text{syn}}}H(t),$$

resulting in a post-synaptic potential

(4) $$\begin{array}{l} {\text{PSP}}_{ij}(t)=\frac{R_{\text{m}}A(J_{ij})e}{\tau_{\text{m}}\tau_{\text{syn}}}(\frac{e^{-t/\tau_{\text{m}}}-e^{-t/\tau_{\text{syn}}}}{{\left(1/\tau_{\text{syn}}-1/\tau_{\text{m}}\right)}^{2}}\\ \;-\frac{te^{-t/\tau_{\text{syn}}}}{1/\tau_{\text{syn}}-1/\tau_{\text{m}}})H(t), \end{array}$$

Here, τ_syn_ is the synaptic time constant, whereas *A*(*J_ij_*) denotes the respective current amplitude needed to evoke a *PSP* of maximal amplitude *J*_*ij*_, cf. Equation (1). *H*(·) denotes the Heaviside function. The current amplitude *A*(*J*) can be computed numerically or in a closed form by using the Lambert-W-function. For fixed *J*_*ij*_, the current amplitude *A*(*J_ij_*) is a function of *R*_m_, τ_m_, and τ_syn_.

**Figure 1:**
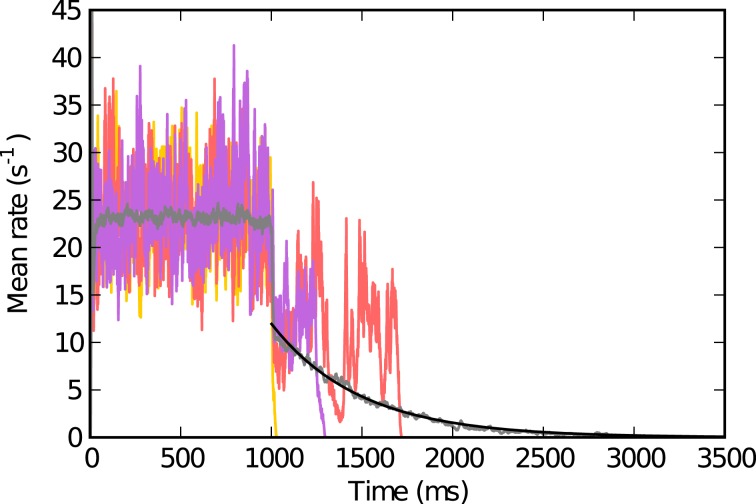
**Population activity in single trials and trial averaged activity**. The individual colored traces (red, yellow, purple) show the population activity while the neurons receive external excitatory input for *t* ∈ [*t*_stim,on_, *t*_stim,off_] (here *t*_stim,on_ = 0 and *t*_stim,off_ = 1000 ms) and after (*t* > *t*_stim,off_) for *g* = 4.4, *J* = 1.1 mV. The trial averaged population activity (averaged over 100 simulations with the same parameters) is depicted in gray. The black curve shows an exponential fit with estimated exponential constant *T* = 487 ms that we define as the lifetime (see text). Other parameters: *N* = 125 000, ϵ = 0.01, *V*_thr_ = 20 mV, *V*_res_ = 0 mV, *R*_*m*_ = 80 MΩ, τ_*m*_ = 20 ms, τ_syn_ = 0.5 ms, τ_ref_ = 2 ms, *d* = 1.5 ms.

**Figure 2:**
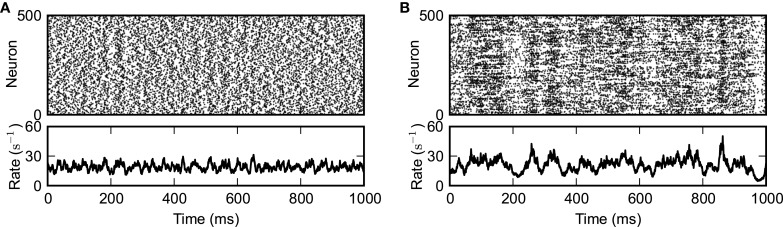
**Externally driven (A) and self-sustained asynchronous-irregular activity (B)**. Spiking activity of a subset of 500 randomly selected neurons (top panels) and instantaneous population-averaged firing rate (“population activity”; bin size 0.5 ms; bottom panels). **(A)** *J* = 0.1 mV, *g* = 4.2, [*t*_stim,on_, *t*_stim,off_] = [−1000, 1000] ms. **(B)** *J* = 1.0 mV, *g* = 4.2, [*t*_stim,on_, *t*_stim,off_] = [−1000, 0] ms. Other parameters as in Figure 1.

**Figure 3:**
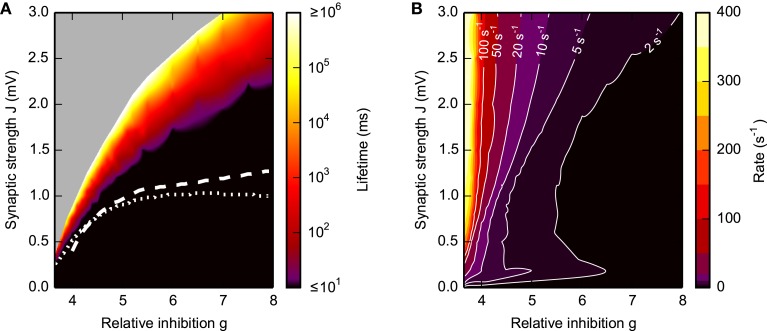
**Lifetime (A) and firing rate (B) of SSAI**. Dependence of the SSAI lifetime **(A)** and mean firing rate **(B)** [cf. Equation (7)] on the synaptic weight *J* and the relative strength *g* of inhibition. Lifetimes and mean firing rates were measured after the external input was turned off. Data represent averages over 10 network realizations. White curves in **(A)** mark saddle-node bifurcations obtained from the diffusion approximation of the LIF neuron [see Brunel, 2000 and Equation S1 in the Supplementary Material with input current mean and variance derived from Equation (17); dotted curve] and from the Abeles-type two-state model (19) (dashed; with *r*^max^ = 1/2τ_ref_, see Section 3.4.1). Other parameters as in Figure 1.

**Figure 4:**
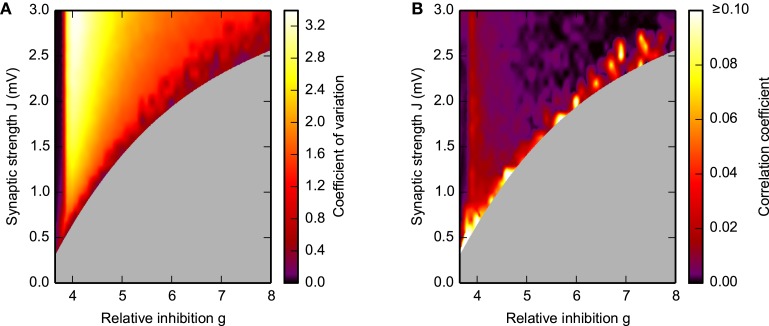
**Spike-train irregularity (A) and pairwise correlations (B) in the SSAI-state**. Dependence of the mean coefficient of variation CV, see Equation (9), of the inter-spike intervals **(A)** and the mean spike-train correlation coefficient Equation (11) **(B)** on the synaptic weight *J* and the relative strength *g* of inhibition. The gray-shaded area marks regions where activity was not sufficient for analysis (see Figure 3A). Other parameters as in Figure 1.

To initially activate the network, in Figures 1–**4**, **10**, **11**, external currents *I*_ext,*i*_(*t*) (*i* ∈ [1, *N*]) are modeled as shot-noise processes (Papoulis and Pillai, 2002) resulting from independent realizations of an inhomogeneous Poisson process with rate

(5) $$\nu_{\text{ext}}(t)=\{\begin{array}{ll} \nu_{\text{ext}} & \text{if }t_{\text{stim,on}}<t\leq t_{\text{stim,off}}\\ 0 & \text{else} \end{array},$$

and a filter kernel as defined by Equation 3. Note that in these cases the external input is only delivered during the period *t* ∈ (*t*_stim,on_, *t*_stim,off_].

**Figure 5:**
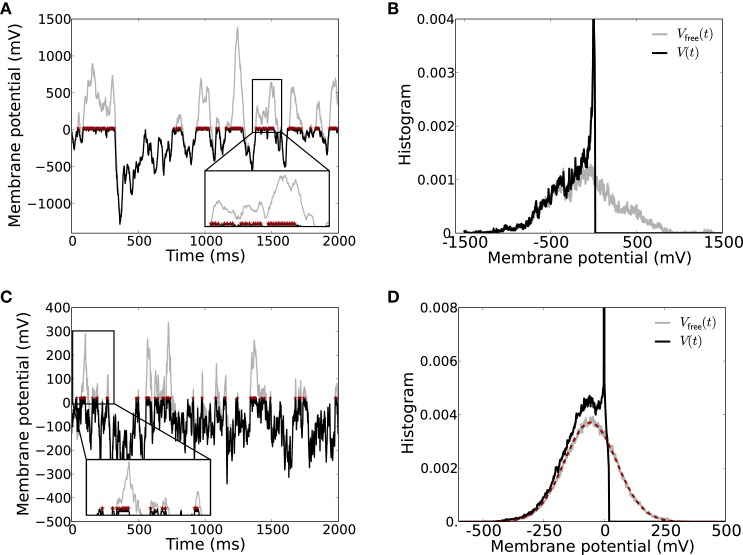
**Strong input fluctuations in strongly coupled networks lead to irregular spiking. (A)** The membrane potential *V*(*t*) including the spike threshold and reset (black) vs. the corresponding free membrane potential *V*_free_(*t*) (gray) recorded from a neuron in a SSAI network. The free membrane potential serves as a monitor of the effective filtered input current the neuron receives. Whenever *V*_free_(*t*) > *V*_thr_, the neuron spikes at high rate *r*∝ 1/τ_ref_ (spikes indicated by red asterisks at the threshold value *V*_thr_). The inset shows a zoom into the membrane potential trace to better show the rapid spiking during suprathreshold fluctuations of the free membrane potential (zoom window *t* ∈ [1370, 1570] ms, *V*(*t*) ∈ [−20, 700] mV). The average spike rate of this neuron was 76.4/s with a CV of 2.91. **(B)** Shows the histogram of the two traces in **(A)**. **(C,D)** Show the same for the reduced Abeles-type model, where the incoming spike trains are assumed to be Poissonian. Here, average spike rate was 36.2/s with a CV of 1.63. The inset zoom window is *t* ∈ [0, 300] ms, *V*(*t*) ∈ [−20, 300] mV. The dashed red curve in **(D)** depicts the expected Gaussian distribution of *V*_free_ with mean and variance given by Equation (17). Parameters: simulation time 40 s, *g* = 4.2, *J* = 3.5 mV, *C_E_* = 400, *C_I_* = 100, other parameters as in Figure 1.

**Figure 6:**
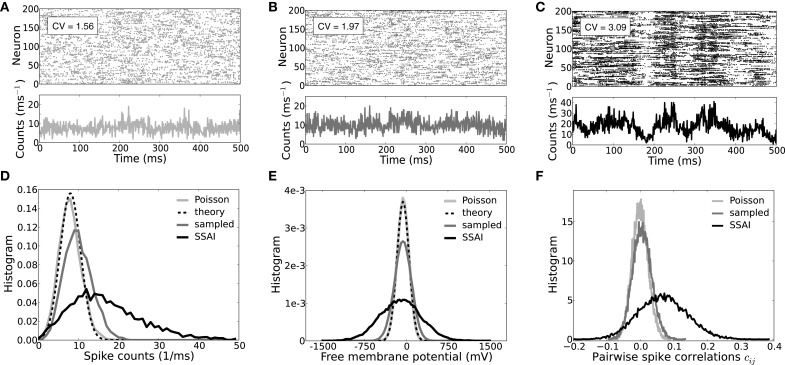
**Spiking dynamics, CVs, free membrane potential, and correlation statistics. (A–C)** Show the spiking activity and population dynamics of 200 neurons in terms of spike counts per millisecond for an ensemble of Poisson-driven LIF neurons (**A**, light gray), an ensemble of neurons in turn driven by spike trains sampled from the ensemble in **(A)** (**B**, dark gray), and the full recurrent SSAI dynamics (**C**, black), respectively. **(D)** Shows the corresponding count distributions, **(E)** depicts the respective distributions of the free membrane potentials, and **(F)** the respective distributions of the pairwise spike train correlation coefficients c_*ij*_. The insets in **(A–C)** show the respective average coefficients of variation CV of interpike-intervals. For otherwise identical parameters the full self-sustained dynamics (**D,E**, black) is characterized by a much more variable population spike count and dynamics of the free membrane potential than the simplified model with Poisson input (**D,E**, light gray) which is fully explained by Equation (23) in **(D)**, and by Equations (16) and (17) in **(E)** (black dashed). **(F)** Illustrates how the pairwise spike train correlations gradually shift to positive values and distributions broaden when neurons sample from the Poisson-driven population (**F**, gray) and in the fully-recurrent SSAI-state (**F**, black) compared to the Poisson-driven ensemble (**F**, light gray), where they cluster around zero. Parameters as in Figure 5.

In **Figures 5C,D**, **6A**, we use external Poisson processes of constant rate filtered by kernels of the form Equation 3 to drive the network over the whole duration of the simulation in order to mimic a network situation with uncorrelated stationary input spike trains, see discussion in Section 3.2.2.

Whenever *V*(*t*) = *V*_thr_, the neuron emits a spike and is reset to *V*(*t*^+^) = *V*_res_ < *V*_thr_. The neuron is then absolute refractory for some time τ_ref_ and clamped at *V*_res_ during this period. We emphasize that even though here synapses with finite time constants are used, all results do not depend on this and generalize, e.g., to networks of neurons with instantaneous δ-shaped synaptic currents. Parameters used in network simulations are specified individually and summarized in Table 2. All simulations were carried out with NEST (Gewaltig and Diesmann, 2007).

### 2.3. Data analysis

#### 2.3.1. Lifetime

For each parameter pair (*g, J*) we performed *k* = 10 simulations with different random realizations of the network. The lifetime *T* of the self-sustained activity is then defined as follows: For each of the 10 network realizations we determine the time *t* at which activity seizes after the external input was turned off at *t*_stim,off_ − *d*, where *d* is the synaptic delay. We find that for given parameters *g* and *J* the survival time of the self-sustained activity after turning off the external input is approximately exponentially distributed (Figure 1). We thus obtain *T* by fitting *e*^−(*t*−*t*_stim,off_)/*T*^ to this data (see Figure 1).

#### 2.3.2. Population rate

The population rate is estimated by the temporal average of the population spike count per time bin Δ*t* = 0.5 ms, i.e.,

(6) $$\nu (t)=\sum_{l=1}^{\left\lfloor t_{\text{tot}}/\Delta t\right\rfloor }\frac{\sum_{i=1}^{N}\chi [i,l]}{\Delta t},$$

where *t*_tot_ is the total time interval under consideration, and χ[*i, l*] is a function that returns the number of spikes of neuron *i* in time bin *l*. To obtain the average firing rate, we compute

(7) $$\bar{\nu }=E\left[\langle \nu (t)\rangle \right]$$

where *E*[.] denotes the average across network realizations and 〈.〉 denotes the temporal average.

#### 2.3.3. Coefficient of variation of inter-spike intervals

To estimate the coefficient of variation (CV) of inter-spike intervals (ISI), we compute the ISI of *n* = 500 neurons, if they spiked at least twice during the time interval *t*_tot_ under consideration. To obtain the average CV, the individual

(8) $$\text{CV}[{\text{ISI}}_{i}]=\frac{\sqrt{\langle {\text{ISI}}_{i}^{2}\rangle -{\langle {\text{ISI}}_{i}\rangle }^{2}}}{\langle {\text{ISI}}_{i}\rangle }$$

are computed and averaged over all neurons *i* ∈ 1, …, *n*, i.e.,

(9) $$\bar{\text{CV}}=\frac{1}{n}\sum_{i=1}^{n}\text{CV}[{\text{ISI}}_{i}].$$

#### 2.3.4. Pairwise correlations

To estimate the pairwise correlations between neurons, we removed the stimulus period *t*_stim_ = *t*_stim,off_ − *t*_stim,on_, see Section 2.3.1, and the initial transient after that stimulus period from the spike train data, that were then binned in time bins of size *h*. *h* was set such that there would be on average 2.5 spikes in each bin, but constrained to *h* ≥ 10 ms. The resulting time series *S_i_*(*t*) were centralized, i.e., the mean was subtracted, such that *S*_*i*_(*t*) = *S_i_*(*t*) − 〈*S_i_*(*t*)〉. Then the auto-covariance functions *A_i_*(τ) = 〈*S*_*i*_(*t*) *S*_*i*_(*t* + τ)〉 and cross-covariance functions *C*_*ij*_(τ) = 〈*S*_*i*_(*t*)*S*_*j*_(*t* + τ)〉 were evaluated at time lag τ = 0. The individual resulting correlation coefficients c_*ij*_ are given by

(10) $${\text{c}}_{ij}=\frac{{\text{C}}_{ij}(0)}{\sqrt{{\text{A}}_{i}(0){\text{A}}_{j}(0)}}.$$

The correlation coefficients c_*ij*_ were computed for a neuron population of size *n* = 500 and then averaged over this subpopulation in order to produce the average correlation coefficient c, i.e.,

(11) $$\bar{\text{c}}=\frac{2}{n(n-1)}\sum_{i=1}^{n}\sum_{j=i+1}^{n}{\text{c}}_{ij}.$$

#### 2.3.5. Network response function

The spiking activity of the network is inherently fluctuating and chaotic. To estimate the response function of the network we thus assume that the instantaneous population rate ν(*t*) at time *t* is a function of the rate ν(*t* − δ*t*) at a previous time *t* − δ*t* plus noise, with δ*t* = 1.5 ms, or analogously

(12) ν(t+δt)−ν(t)=Δν(ν(t))+ξ(t),

where the noise ξ(*t*) is assumed to be a stationary process. To estimate the response function Δν(ν), the instantaneous network rate, calculated in time bins *t_i_* of size Δ*t* = 0.5 ms, was binned into *n_b_* = 40 bins of equal size δν, and for each bin ν_*j*_, the average response was calculated as

(13) $$\Delta \nu (\nu_{j})={\langle \nu (t_{i}+\delta t)\rangle }_{\nu (t_{i})\in \nu_{j}}-\nu_{j},$$

where the average is taken over all *i* such that ν(*t_i_*) was in the bin centered on ν_*j*_, i.e., $\nu_{j}-\frac{1}{2}\delta \nu \leq \nu (t_{i})<\nu_{j}+\frac{1}{2}\delta \nu$. The data from *k* = 10 simulations with different random realizations of the network was aggregated into one average response function.

### 2.4. Abeles model

In many simplified integrate-and-fire neuron models that receive temporally fluctuating input current from a pool of presynaptic neurons, the probability to emit a spike is determined by two key properties of this integrated input: its mean and variance with respect to the firing threshold. In essence, the output rate of such a neuron will depend on the probability that the free membrane potential is suprathreshold.

This is the essence of models as proposed in Abeles (1982) and Amit and Brunel (1997). The membrane potential distribution in absence of a threshold (free membrane potential *V*_free_) can often be approximated by a Gaussian

(14) $$P(V_{\text{free}},\mu ,\sigma )=\frac{1}{\sqrt{2\pi }\sigma }e^{\frac{-{\left(\mu -V_{\text{free}}\right)}^{2}}{2\sigma^{2}}},$$

where μ = μ[*V*_free_] and σ = σ[*V*_free_] are the mean and standard deviation of the free membrane potential. The area under the Gaussian above firing threshold can then be related to the firing probability *f*(μ, σ) in the following way:

(15) $$f(\mu ,\sigma )=\frac{1}{\sqrt{2\pi }\tau }\int_{\frac{V_{\text{thr}}-\mu }{\sigma }}^{\infty }e^{-x^{2}/2}dx,$$

where τ denotes a characteristic memory time constant, e.g., the membrane time constant.

## 3. Results

We investigate the transition in the dynamic behavior that random networks of inhibitory and excitatory LIF neurons undergo when the synaptic coupling strength *J* is increased. For small *J*, the network needs permanent external drive to remain active (Brunel, 2000). Depending on the strength of this external drive and the synaptic coupling parameters *g* and *J*, spiking activity can be asynchronous and irregular (Figure 2A). For sufficiently large *J*, however, the network can stay active even in the absence of external drive, i.e., for *I*_ext_ = 0. Spiking is much more irregular in this self-sustained regime and population activity is characterized by pronounced fluctuations (Figure 2B). In the present paper, we investigate the mechanisms underlying the emergence, the spike-train irregularity, and the lifetime of self-sustained asynchronous-irregular (SSAI) activity.

### 3.1. Characteristics of self-sustained activity in random LIF networks with strong synapses

To characterize the dynamical features of the SSAI state, we first analyze the lifetime, firing rate, irregularity and correlations in dependence of coupling strength *J* and relative inhibition *g*, here for network size of *N* = 1.25× 10^5^ with connection probability ϵ = 0.01.

The lifetime of the SSAI increases rapidly from zero to more than 1000 s (i.e., networks stay active for the whole duration of the simulation) within a narrow band in the parameter space spanned by *g* and *J*, see Figure 3A. This transition band becomes wider, i.e., more gradual in terms of *J*, as *g* is increased, indicating a more shallow transition between transient and stable self-sustained activation. The rate of the persistent activity is typically between 20 and 50 s^−1^, increasing to 400 s^−1^ when excitation becomes dominant at *g* < 4, see Figure 3B.

Figure 4A, moreover, demonstrates that during SSAI the coefficient of variation (CV) of inter-spike interval (ISI) are typically substantially higher than unity, meaning that spike trains are more irregular than a Poisson process, while Figure 4B shows that pairwise spike-train correlations—indicating residual synchrony—decrease for longer lifetimes, especially in the region of large *g* and *J*.

In summary, for wide regions of the *g*-*J*-parameter space, network activity is sustained without external drive for long time periods, the firing rates are in an intermediate range and spiking activity is highly irregular and asynchronous. In the next section, we suggest a simple mechanism for the emergence of SSAI.

### 3.2. Basic mechanism underlying self-sustained asynchronous-irregular activity

Several earlier studies suggested that the self-sustained asynchronous-irregular activation we observe here is impossible in balanced random networks with current-based synapses (Kumar et al., 2008; El Boustani and Destexhe, 2009). To resolve this apparent contradiction, we now analyze the membrane potential dynamics in the SSAI-state. This will lead us to a reduced Abeles-type model, cf. Equation (15), that demonstrates the basic mechanism, i.e., the trade-off between the mean and the variance of the input of the neurons, underlying the occurrence of self-sustained activity.

#### 3.2.1. Large membrane potential fluctuations induce highly irregular spiking

Inspection of the membrane potential traces of neurons in SSAI states reveals that they fluctuate strongly (on the order of volts, rather than millivolts, depending on the amplitude of input current variance), only limited by the threshold for positive values and the maximally possible inhibitory input for negative values, which depends on the dynamical state of the system.

If we consider the free membrane potential *V*_free_(*t*), i.e., the membrane potential dynamics if the spike threshold *V*_thr_ is set to infinity, as a representative monitor for the filtered input from the network, we see that *V*_free_(*t*) also has large excursions to positive values, cf. Figure 5A, gray curve. The corresponding normalized histograms for these particular traces are shown in Figure 5B. Note that for the neuron model with finite spike threshold (black) the membrane potential cannot be beyond threshold *V*_thr_, and instead there is a large peak in the histogram around the reset potential *V*_res_ (the amplitude of the peak is approximately 0.056, not shown).

Moreover, due to these extreme fluctuations the neuron reset amplitude becomes almost negligible due to the occasional massive net-excitatory input transients, and as long as the free membrane potential *V*_free_(*t*) is above threshold *V*_thr_ and has positive derivative, the neuron fires at close to the maximum rate given by *r*^max^ ~ 1/τ_ref_ (see inset in Figure 5A for illustration). The free membrane potential must have positive derivative, i.e., the neuron must receive net excitatory current, to drive the neuron to threshold because of the subthreshold reset after the spike. A large fraction of time, however, the membrane potential spends far below the threshold, leading to long periods of time where the neuron does not spike. This results in highly irregular spike trains with coefficients of variation (CV) larger than unity (here, *CV* = 2.91).

#### 3.2.2. LIF-neuron driven by strongly weighted poisson input

The full self-consistent dynamics of self-sustained activity states is hard to assess because of the non-linear input-output relation of LIF neurons and the non-Poissonian nature of the compound input spike trains that characterizes the SSAI-state. To address the spiking irregularity in the case of strongly weighted input spikes, we thus now consider a simplified scenario where we assume that the incoming spike trains are independent stationary Poisson processes, implying a CV of unity for the input spike trains.

Already in this case, *V*_free_(*t*) spends large fractions of time at very hyperpolarized values, and only occasionally there are suprathreshold fluctuations, resulting in long periods of silence, interrupted by burst-like spiking, see Figure 5C. The distribution of *V*_free_(*t*) (Figure 5D) is narrower than for the full recurrent dynamics shown in Figures 5A,B, yet already covers several hundred millivolts. The simple structure of the Poisson input, moreover, allows to derive the distribution of *V*_free_(*t*) (red dashed curve in Figure 5D) as we will discuss in the next section.

The spiking activity, coefficient of variation CV, population spike count, free membrane statistics, and pairwise spike train correlation coefficient c_*ij*_ of uncoupled LIF neurons driven by such approximately balanced, but strongly weighted Poisson input, are shown in Figures 6A,D–F (light gray). Indeed, even in this reduced model the average CV of the output spike train-ISI is beyond unity at CV ~ 1.6, i.e., spiking is more irregular than Poisson (Figure 6A). Pairwise spike train correlations were computed for 500 randomly selected neurons. As to be expected for uncoupled neurons injected with uncorrelated Poisson input, correlation coefficients are symmetrically distributed around zero, cf. Figure 6F.

Figure 6B shows the spiking and population count activity for 200 LIF neurons with input spike trains sampled from the Poisson-driven population shown in Figure 6A, with the same common input structure as in the recurrent network (first-order recurrence). The corresponding CV ~ 2, population count distribution, and free membrane potential distribution (Figures 6B,D,E, dark gray lines) show that variability is greater than for the Poisson-driven case, but still much smaller than in the full SSAI dynamics (Figures 6A,C–E, black). Spike train correlations are now slightly positive on average, here c = 5.4 × 10^−3^, yet correlation coefficients are still approximately symmetrically distributed around zero.

In Figure 6C the corresponding full self-consistent SSAI dynamics for identical parameters is shown (∞-order reccurence), revealing the higher amplitude of population fluctuations and spiking variability with an average CV of spike train-ISI of CV ~ 3. The population spike count is skewed to higher values, see black line in Figure 6D, indicating the increased transients of correlated spiking that are visible as vertical stripes in the spike raster plot in Figure 6C. Indeed, spike train correlations (see Figure 6F, black line) are now clearly positive on average with a more than ten-fold increased value of c = 0.068 compared to the correlations between spike trains shown in Figure 6B.

This demonstrates how the full recurrent network amplifies weak pairwise correlations and irregularity of spiking, yielding much larger population fluctuations, wider free membrane potential distribution, and higher CV of ISIs compared to what is expected from the Poisson-input assumption. Moreover, as the variability increases, also firing rates increase. For the Poisson-driven ensemble the average rate is 36 s^−1^ (Figure 6A), for the ensemble-sampling neurons it is 51.1 s^−1^ (Figure 6B), and for the full self-sustained dynamics it is 81 s^−1^ (Figure 6C). At the same time, the fraction of ISIs that are close to the minimal ISI τ_ref_ = 2 ms becomes larger. If we denote the interval between τ_ref_ and τ_ref_ + 1 ms by ISI_1_, and the next ISI_2_ := [τ_ref_ + 1 ms, τ_ref_ + 2 ms], the fraction *f*[ISI] of ISIs falling into these bins are (*f*[ISI_1_], *f*[ISI_2_]) = (0.14, 0.15) for spike trains in Figure 6A, (*f*[ISI_1_], *f*[ISI_2_]) = (0.22, 0.23) for spike trains in Figure 6B, and (*f*[ISI_1_], *f*[ISI_2_]) = (0.54, 0.2) for spike trains in Figure 6C. This means, that while only about 30% of ISIs are shorter than 4 ms for neurons sampling from Poisson input, about 75% of ISIs in the recurrent SSAI-network fall into this category.

#### 3.2.3. Reduced two-state Abeles-type firing rate model

From the observations of the last two sections, we will now derive a simple dynamical model to analyze the basic mechanism underlying the saddle-node bifurcation that leads to the emergence of a second stable fixed-point at finite rate, i.e., the self-sustained state. As discussed in Section 3.2.2, if *J* is strong, the resulting membrane potential of a LIF neuron undergoes large fluctuations also in the case of strongly weighted uncorrelated Poisson-input. Spikes are emitted at high rate *r* whenever the free membrane potential, i.e., the effective neuron drive, is (i) above threshold and (ii) has positive derivative, while the neuron is quiescent at basically all other times, cf. Figure 5C. The free membrane potential fluctuates around a fixed mean, and if the input is approximately balanced, the derivative of *V*_free_(*t*) should be positive about half the time, i.e., we estimate the firing rate during suprathreshold excursions to be *r* ≲ *r*^max^ =: 1/2τ_ref_.

To derive the time that *V*_free_(*t*) is in the suprathreshold state, we observe that for uncorrelated stationary Poisson inputs of rate ν_*j*_ the distribution of the free membrane potential *V*_free_ is approximately given by a Gaussian with mean μ = μ[*V*_free_] and standard deviation σ = σ[*V*_free_], such that

(16) $$P(V_{\text{free}},\mu ,\sigma )=\frac{\text{exp}\left[\frac{-{\left(V_{\text{free}}-\mu \right)}^{2}}{2\sigma^{2}}\right]}{\sqrt{2\pi }\sigma }$$

with

(17) $$\begin{array}{l} \mu [V_{\text{free}}]=\sum_{j\in \text{Pre}[i]}\nu_{j}\int_{0}^{\infty }{\text{PSP}}_{ij}(t)dt,\sigma^{2}[V_{\text{free}}]\\ \;=\sum_{j\in \text{Pre}[i]}\nu_{j}\int_{0}^{\infty }{\text{PSP}}_{ij}^{2}(t)dt. \end{array}$$

The PSP(*t*) for α-type synapses is defined in Equation (4).

The probability *q*_>*V*_thr__ for the free membrane potential to be above threshold thus equals the fraction of the area under *P*(*V*_free_, μ, σ) above the threshold, i.e.,

(18) $$q_{>V_{\text{thr}}}(\mu ,\sigma )=\frac{1}{2}\left(1-\text{erf}\left[\frac{V_{\text{thr}}-\mu }{\sqrt{2}\sigma }\right]\right).$$

All neurons in expectation spike at the same rate, such that Equation (18) can in analogy to the Abeles model Equation (15) be used to estimate an upper bound 〈ν(*t*)〉^max^ for the time-averaged firing rate of the neuron, if we assume that the neurons keep integrating inputs while in the refractory state^1^, i.e.,

(19) $${\langle \nu (t)\rangle }^{\text{max}}=q_{>V_{\text{thr}}}(\mu ,\sigma )\times r^{\text{max}}=\;\frac{q_{>V_{\text{thr}}}(\mu ,\sigma )}{2\tau_{\text{ref}}}.$$

Because μ and σ are functions of ν(*t*), we can find the self-consistent rate solution for any given parameter set {*J, g, C_E_, C_I_*}, i.e.,

(20) $$\nu_{0}=q_{>V_{\text{thr}}}(\mu_{0},\sigma_{0})\times r,$$

where μ_*o*_ = μ(ν_0_) and σ_0_ = σ(ν_0_) again are the self-consistent mean and standard deviation.

Moreover, we can assess the critical parameters for which (i) there exists a 〈ν(*t*)〉 = ν_0_, such that Equation (20) has a self-consistent solution, and (ii) this solution is stable. The latter is determined by computing the slope of Equation (19) at ν_0_, i.e.,

(21) $$\begin{array}{l} {\left|\frac{d\left(q_{>V_{\text{thr}}}(\langle \nu (t)\rangle )\times r\right)}{d\langle \nu (t)\rangle }\right|}_{\langle \nu (t)\rangle =\nu_{0}}=\\ \;\left|\frac{r}{\nu_{0}}\frac{\left(\mu_{0}+V_{\text{thr}}\right)}{\sqrt{8\pi }\sigma_{0}}e^{-\frac{{\left(V_{\text{thr}}-\mu_{0}\right)}^{2}}{2\sigma_{0}^{2}}}\right|\overset{!}{<}1, \end{array}$$

where the final condition is necessary for stability.

**Figure 7:**
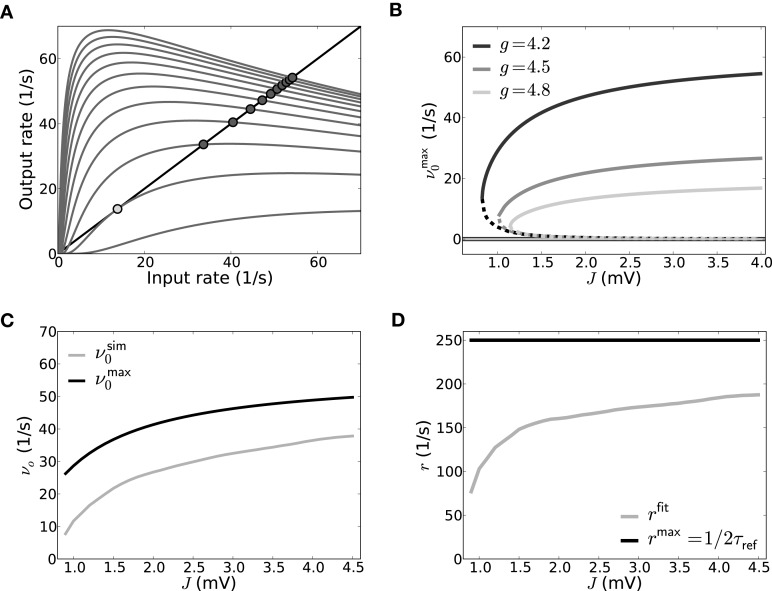
**Self-consistent rates as a function of coupling strength in the reduced model assuming Poisson input spike trains. (A)** The reduced Abeles-type model allows for a straight-forward evaluation of the rate fixed-point, its emergence and stability. ν^max^_0_ := *q*_>*V*_thr__(ν)/2τ_ref_ as function of ν is shown here for different values of *J* (*J* = {0.525, 0.825, …, 3.825} from bottom to top and *g* = 4.2), the intersections of the curves with the diagonal line mark the fixed-points; in particular the coalescence point is marked by a light gray circle, the stable non-trivial fixed-points by dark gray circles). The zero-rate fixed-point and unstable fixed-point are not explicitly marked for sake of visibility. **(B)** Shows all three fixed-point states ν^max^_0_ as a function of *J* for three different *g*, where the solid lines denote the stable fixed-points at zero rate and at high rates, while the dashed line denotes the unstable intermediate fixed-point. **(C)** shows the self-consistent high rate fixed-point for a network where input spike trains are Poissonian. The solid gray line shows the self-consistent rate as obtained from a direct simulation of the simplified model, and the solid black line shows the rate as predicted from Equation (20) with *r*^max^ = 1/2τ_ref_ (*g* = 4.2, cf. **B**). In **(D)** *r*^fit^, the rate fitted to match the result for ν^sim^_0_ from the direct simulation, is depicted as a function of coupling strength (gray) and compared to *r*^max^ (black). Parameters as in Figure 5.

Figure 7A demonstrates this saddle-node bifurcation by evaluating the output rate Equation (19) with μ and σ as a function of input rate ν for increasing *J* and *g* = 4.2. The crossing of the resulting curve (gray) with the bisection line (black) indicates identity of input and output, i.e., fixed-points. The saddle-point that marks the onset of the saddle-node bifurcation is depicted in light gray, while the resulting new stable high-rate fixed-points after the saddle-node bifurcation are marked by dark gray dots.

For increasing *J*, the new intermediate unstable fixed-point moves closer to the zero-rate fixed-point. This is shown in Figure 7B which depicts the dependence of the fixed-points of ν^max^_0_ := *q*_>*V*_thr__(ν_0_)/2τ_ref_ on *J* for three different *g*: For increasing *J* the resulting high-rate fixed-points (Figure 7B, non-zero solid lines) of ν^max^_0_ first quickly increase, but eventually level out, in line with the closer spacing we observe in Figure 7A. The intermediate unstable fixed-points (Figure 7B, dashed lines) move to smaller rates for increasing coupling strength *J*, asymptotically moving toward the zero-rate fixed-point. This indicates a loss of stability of the zero-rate fixed-point with increasing coupling strength. This is akin to the situation in the full spiking system where a single spike– the smallest perturbation from the quiescent state– can suffice to activate the SSAI state, if *J* becomes of the order of the distance between resting and threshold potential, see Supplementary Material Section 3. Finally, for fixed *J*, both intermediate and high-rate fixed-point rates decrease with increasing inhibition *g* (from dark to light gray).

In the Abeles model, a smaller fixed-point rate corresponds to a smaller area of the free membrane potential above threshold, i.e., smaller *q*_>*V*_thr__(ν). The area above threshold is determined by the trade-off between mean and standard-deviation: for fixed mean μ[*V*_free_], an increase in σ[*V*_free_] can increase the area above threshold, while for fixed σ[*V*_free_] the mean μ[*V*_free_] will determine, if and how much mass of the free membrane potential distribution is suprathreshold. The mean of *V*_free_ in our networks is typically negative (*g* ≥ 4), such that σ[*V*_free_] should be of the order of (*V*_thr_ − μ[*V*_free_]) to have a significant contribution in *q*_>*V*_thr__(ν), see Equation (18).

For example, evaluation of Equations (17) shows that μ[*V*_free_] is linearly dependent on *C_E_*, *C_I_* and 〈ν(*t*)〉, while the standard deviation has a square-root dependence instead, such that a change in any of these parameters can lead to a faster decrease in mean than increase in standard deviation. Moreover, even though both mean and standard variation are linear in the respective synaptic current amplitude *A*, and thus in *g* and *J*, inspection of Equation (17) shows that the mean outweighs the standard deviation quickly if the rate ν is not too small and *g* is not close to *C_E_*/*C_I_*. This predicts that in these cases no self-consistent solutions may exist.

From the full spiking network, however, we saw that there is a wide range of *g* and *J* values that lead to long periods of sustained activity (see Figure 3A). We hypothesize that this is innately related to the large population variance, and thus also input current variance, and the spiking irregularity in these self-sustained systems. The variance of the free membrane potential *V*_free_(*t*) is already larger, if a neuron samples from the Poisson-driven ensemble, cf. Figure 6D, and some of the increased variance is hence explained by the more irregular input spike statistics. Counterintuitively, larger population activity fluctuations and spiking irregularity can thus make the system more likely to sustain spiking activity in the absence of external input by increasing the likelihood for suprathreshold input transients.

To test how well the reduced two-state approach performs compared to actual spiking neurons, we simulated a population of LIF neurons with balanced Poisson inputs to mimic a network of size *N* = 5000 with connection probability ϵ = 0.1 and a ratio between excitation and inhibition of four, i.e., it received *C_E_* = 400 excitatory and *C_I_* = 100 inhibitory input spike trains.

In order to mimic the self-consistent state, these Poisson inputs had a rate ν_0_ = ν^sim^_0_ that was numerically tuned such that the *N* stimulated neurons on average spiked with ν_0_ themselves. ν^sim^_0_ is smaller than what is predicted by Equation (20) with *r*^max^ = 1/2τ_ref_, cf. Figure 7C. Indeed, when we solved for the corresponding spike rate *r* = *r*^fit^ in Equation (20) that in turn resulted in ν^sim^_0_, we found it to be generally smaller than 1/2τ_ref_ for the parameters chosen here. Also, it depends on the average firing rate and coupling strength in that it gets closer to 1/2τ_ref_ for larger ν^sim^_0_ and *J*, cf. Figure 7D. The discrepancy is mostly due to the fact that it takes neurons a finite time to move back to firing threshold after emitting a spike in the presence of fluctuating input currents, and this effect is stronger for smaller fluctuation amplitudes. We remark that for the full SSAI-network shown in Figure 6C, Equation (20) gives the right quantitative rate, if evaluated with *V*_free_ measured from the simulation. The good agreement is explained by the much higher fraction of short ISIs reported in Section 3.2.2, justifying the assumption of *r* = 1/2τ_ref_.

Within the simplified two-state Abeles model approach followed here, we cannot only derive the self-consistent firing rate, but also the approximate distribution of the population spiking activity. The probability for any neuron to be in the active state and fire with rate *r* is given by *q*_>*V*_thr__(ν_0_). Thus, the probability *B*(*k*|*N, q*_>*V*_thr__(ν_0_)) to have *k* active neurons in an ensemble of *N* identical neurons is given by the binomial distribution

(22) $$B(k|N,q_{>V_{\text{thr}}}(\nu_{0}))=\left(\begin{array}{c} N\\ k \end{array}\right)q_{>V_{\text{thr}}}{\left(\nu_{0}\right)}^{k}{\left(1-q_{>V_{\text{thr}}}(\nu_{0})\right)}^{N-k}.\;\;\;\;$$

The expected number and variance of counts in a time bin Δ*t* is then given by

(23) $$\begin{array}{l} \;E[\text{counts}]=Nq_{>V_{\text{thr}}}(\nu_{0})r\Delta t,\\ \text{Var}[\text{counts}]=Nq_{>V_{\text{thr}}}(\nu_{0})(1-q_{>V_{\text{thr}}}(\nu_{0}))r\Delta t. \end{array}$$

We indeed find very good agreement for Poisson-driven LIF neurons with ν_0_ = ν^sim^_0_ and *r* = *r*^fit^, see Figure 6D.

The two-state firing rate approximation for Poisson-driven LIF neurons is thus a valuable tool to gain qualitative insight into the basic mechanisms that underlie SSAI in random networks of excitatory and inhibitory spiking neurons.

### 3.3. Effect of coupling strength heterogeneity on the emergence of SSAI in the two-state Abeles-type model

So far we considered networks where all excitatory synapses are weighted by the same weight *J*, and all inhibitory synapses by the same weight −*gJ*, respectively, and studied how the emergence of SSAI depends on these parameters, both in explicit simulations, as well as in the two-state firing rate Abeles-type model. Yet, in this reduced firing rate framework it is straightforward to investigate the impact of arbitrary parameters on the emergence of SSAI, in particular the effect of more realistic weight distributions with finite variance.

If we assume that all synaptic weights are distributed according to some excitatory and inhibitory weight distribution *P*(*W_iE_*) and *P*(*W_iI_*), respectively, the variance of the free membrane potential is given by

(24) $$\begin{array}{l} \sigma_{i}^{2}[V_{\text{free}}]={\text{E}}_{W}[\sum_{j\in \text{exc}}\nu_{j}W_{ij}^{2}\int_{0}^{\infty }{\text{PSP}}_{ij}^{2}(t)dt\\ \;+\sum_{j\in \text{inh}}\nu_{j}W_{ij}^{2}\int_{0}^{\infty }{\text{PSP}}_{ij}^{2}(t)dt]\\ \;=\epsilon_{iE}N_{E}\alpha_{iE}\nu_{E}{\text{E}}_{W}\left[W_{iE}^{2}\right]+\epsilon_{iI}N_{I}\alpha_{iI}\nu_{I}{\text{E}}_{W}\left[W_{iI}^{2}\right]\\ \;\geq \epsilon_{iE}N_{E}\alpha_{iE}\nu_{E}{\text{E}}_{W}{\left[W_{iE}\right]}^{2}+\epsilon_{iI}N_{I}\alpha_{iI}\nu_{I}{\text{E}}_{W}{\left[W_{iI}\right]}^{2}, \end{array}$$

with expectation value across network realizations E*_W_*[.], α_*iX*_ := ∫^∞^_0_PSP^2^_*iX*_(*t*)*dt*, *X* ∈ {*I, E*}, cf. Equation (4), and ν_*I*_, ν_*E*_ are the firing rates of the inhibitory and excitatory neurons, which for simplicity we assume to be stationary and the same for all neurons of one type. Note, that the PSP(*t*) without loss of generality are now normalized such that their peak-amplitude equals 1 mV, and the *W*_*ij*_ are dimensionless numbers. So as to be expected, because E*_W_*[*W*^2^_*ij*_] ≥ E*_W_*[*W_ij_*]^2^ any finite variance of the weight distribution will increase the input current distribution variance as well.

Many experimental studies report lognormally distributed synaptic weights *W*_*ij*_ ~ Log-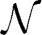 (*m, s*) (Song et al., 2005; Lefort et al., 2009; Avermann et al., 2012), i.e., the logarithm of the weights Log[*W_ij_*] is normally distributed. Such distributions are parametrized by *m* and *s*, i.e., the mean and standard deviation of the distribution of Log[*W_ij_*]. The mean and raw variance of the lognormally distributed weights are then given by

(25) $${\text{E}}_{W}[W_{ij}]=e^{m+s^{2}/2}\text{and }{\text{E}}_{W}[W_{ij}^{2}]=e^{2\left(m+s^{2}\right)}.$$

For this type of weight distribution we obtain

(26) $$\sigma_{i}^{2}[V_{\text{free}}]=\epsilon_{iE}N_{E}\alpha_{iE}e^{2\left(m_{E}+s_{E}^{2}\right)}\nu_{E}+\epsilon_{iI}N_{I}\alpha_{iI}e^{2\left(m_{I}+s_{I}^{2}\right)}\nu_{I}.$$

**Figure 8:**
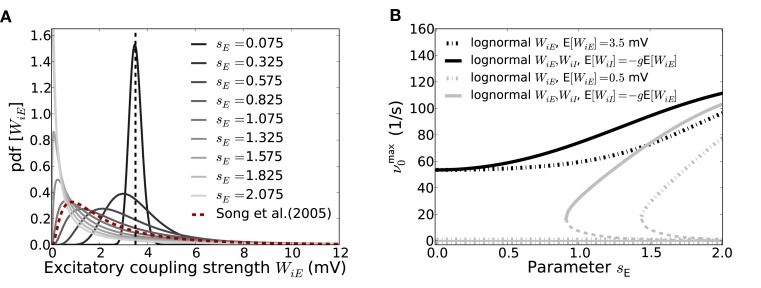
**Effect of the weight distribution on the SSAI state in the reduced Abeles-type model. (A)** Shows the excitatory weight distribution as a function of the lognormal parameter *s_E_* for fixed expectation value E*_W_*[*W_iE_*] (indicated by the vertical dashed line). Even though the mean coupling strength is thus the same, the median moves to the left and the variance increases for increasing *s_E_*, such that most synapses are very weak, but few are very strong. For comparison, we also plot the EPSP-distribution found for layer 5 pyramidal cells in visual cortex by Song et al. (2005) (red dashed line). It proves compatible with the curve for *s_E_* = 1.325, showing that such values are not unrealistic for cortical networks. **(B)** demonstrates the effect of increasing *s_E_* on the saddle-node bifurcation point. Solid lines mark the high rate stable fixed-point rate for the SSA state as predicted from the two-state Abeles-type model for *W*_*ij*_ ~ Log-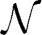 (*m, s*) with parameters *m*, *s* chosen such that the mean coupling strengths are constant at E*_W_*[|*W_iE_*|] = *J* = 3.5, E*_W_*[|*W_iI_*|] = *gJ* = 4.2*J* (black lines) and E*_W_*[|*W_iE_*|] = *J* = 0.5, E*_W_*[|*W_iI_*|] = *gJ* = 4.2*J* (gray lines), respectively. The solid lines denote the case where only excitation is distributed lognormally, while all inhibitory weights are −*gJ* (*s_I_*≡ 0), while the dash-dotted lines denote the case where also inhibition is distributed lognormally with *s_I_* = *s_E_*; *m_E_* = Log[*J*] − *s*^2^*_E_*/2 and *m_I_* = Log[*gJ*] − *s*^2^*_I_*/2, respectively. For *J* = 3.5 the high-rate fixed-point exists for all *s_E_*, independent of the variance of the inhibitory weight distribution. The zero-rate and unstable intermediate fixed-points close to zero (see Figure 7B) are not included. For *J* = 0.5 we observe a saddle-node bifurcation for increasing *s_E_* that occurs earlier if inhibitory weights are also lognormally distributed. The intermediate fixed-points are denoted by the dashed lines. All other parameters as in Figure 5.

How this increased input variance in terms of the parameters *m, s* of the lognormal weight distribution affects the emergence and fixed-point firing rate of the SSAI-state for the Abeles-type model, cf. Section 3.2.3, is shown in Figure 8.

If we fix the average values of the excitatory and inhibitory coupling strengths E*_W_*[|*W_iE_*|] = *J* and E*_W_*[|*W_iI_*|] = *gJ*, respectively (and thus μ[*V*_free_]), a lognormal distribution has left one effective degree of freedom. If we decide to vary the width-parameter *s*, the respective *m* must be *m* = Log[*W*] − *s*^2^. So, if E*_W_*[*W*] is fixed, larger *s* implies smaller *m*. The median of the lognormal distribution is given by *e*^*m*^, such that for decreasing *m* more and more of the total number of synapses will have very small weight, while a small number will have very large weight, and the total variance grows. Figure 8A shows the resulting effect of increasing *s_E_* on the weight distribution. The larger *s_E_* becomes (from dark to light gray), the more skewed and heavy-tailed the weight distribution gets for same mean coupling strength (denoted by the black dashed line). For comparison, we also plot the weight distribution as reported in (Song et al., 2005) (red line), where the authors measured EPSP-amplitudes between layer 5 pyramidal cells from visual cortex. The resulting curve is compatible with *s_E_* ≈ 1.32 for the chosen *J* = 3.5 mV. In fact, the expectation value of the data curve is E[*W_ij_*] = 3.13 mV, which is of the same order as the average weight chosen here. Other studies report lognormal weight distributions with expectation values of the order of E[*W_ij_*] = 0.5 mV for unitary EPSP- and IPSP-amplitudes in layers 2/3 of the mouse barrel cortex (Lefort et al., 2009; Avermann et al., 2012).

The key effect of increasing the variance of the free membrane potential in this way, while keeping the mean fixed, is a decrease in the critical average coupling strength for the saddle-node bifurcation to occur. This is exemplified in Figure 8B: The black lines show the high-rate fixed-point rate for E[*W_iE_*] = 3.5 and E[*W_iI_*] = −*gE*[*W_iE_*], with *g* = 4.2, for varying *s_E_* and zero *s_I_* (dash-dotted line), and both varying *s_E_* = *s_I_* (solid line). For this average coupling strength, the network is beyond the saddle-node bifurcation even for zero variance of the weight distribution, cf. Figure 7B, so in both cases the lines start at non-zero rate for *s_E_* = 0. The main effect of increasing *s_E_* is thus an increase in fixed-point rate, explained by the increased variance of the free membrane potential distribution for same expectation value, i.e., larger *q*_>*V*_thr__(ν).

The gray lines show the same setup for E*_W_*[*W_iE_*] = 0.5 mV. In this case, the zero-variance distribution analysis of Equation (20) predicts that there is only the zero-rate fixed-point. With increasing finite variance *s_E_* the system undergoes a saddle-node bifurcation, see gray curves in Figure 7B. Moreover, because of the larger variance, this bifurcation happens earlier for the case where both excitatory and inhibitory weights have finite variance *s_E_* (solid line), but exists as well for the case where inhibitory weights are all identical (see also Teramae et al., 2012; Ikegaya et al., 2013).

Similar effects are expected from every manipulation that increases the variance of the free membrane potential, while keeping the mean approximately fixed, as well as manipulations increasing the mean for fixed or increasing variance, e.g., by varying the number of synaptic inputs *C_E_*, *C_I_* = γ*C_E_* for fixed weights, as well as changing the amount of relative inhibition by γ or *g*.

### 3.4. Lifetime of SSAI states in a stochastic rate model

So far we analyzed the occurrence, variability and irregularity in terms of a reduced two-state Abeles-type model. But can we understand the transition from finite to virtually infinite lifetimes in the fully recurrent networks when the synaptic coupling strength increases?

**Figure 9:**
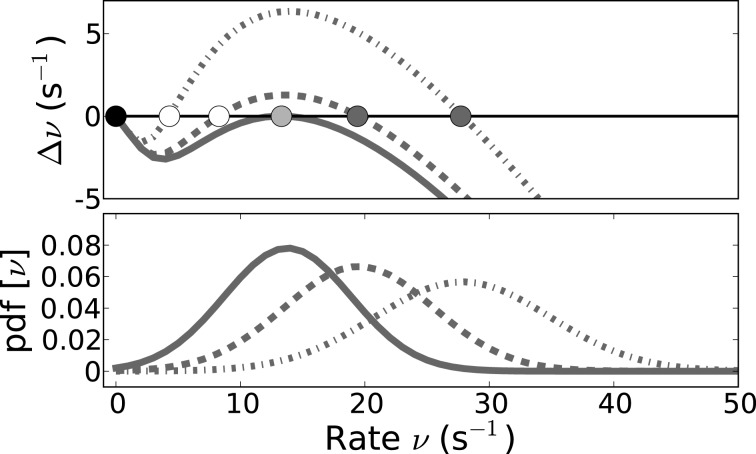
**The lifetime of SSAI states is determined by the size of population fluctuations vs. the size of basin of attraction**. The upper panel shows the emergence of the saddle-node bifurcation that underlies the SSAI state (coalescence point for critical coupling strength *J_c_* depicted in light gray), while the lower panel shows the population rate distributions for neurons that are driven by uncorrelated Poisson inputs, for three different values of *J* each (derived from Equation (23) by translating the counts to rates and approximating the binomial by a Gaussian, solid lines: *J* = *J*_*c*_ = 0.641 mV, dashed *J* = 0.651 mV, dashed-dotted *J* = 0.731 mV). In the upper panel the stable high-rate fixed-points for *J* > *J*_*c*_ are marked by dark gray, the unstable intermediate fixed-point by white circles. The black circle indicates the trivial zero-rate fixed-point. In the SSAI state the system constantly produces large population fluctuations that can drive the system substantially far away from the high-rate fixed-point. We expect the system to become stable to this inherently generated fluctuations when the basin of attraction (here the distance between the unstable and stable fixed-point) becomes larger than the characteristic size of the fluctuations, given by the variance of the population rate distribution. Parameters as in Figure 5.

As shown in the previous sections large population-rate variability is an inherent feature of self-sustained activity states. So the system perpetually perturbs itself and can substantially deviate from the high rate fixed-point ν_0_. If the basin of attraction is smaller than the characteristic fluctuation size, the system can escape the attractor and run into the trivial attractor at zero rate. Inspection of

(27) $$\Delta \nu (\nu ):=q_{>V_{\text{thr}}}(\nu )\times r^{\text{max}}-\nu ,$$

cf. Equation (19), as a function of the input rate ν (Figure 9, upper panel) reveals the basin of attraction of the high-rate fixed-point as the interval between the unstable (indicated by white circles) and the stable (dark gray circles) fixed-points that are the zeros for ν > 0 of Equation (27). The black circle represents the zero-rate fixed-point ν = 0.

The upper panel in Figure 9 shows the respective curves for three different values of *J*, with all other parameters fixed. The lower panel shows the distribution of the population activity, as predicted from Equation (22) with the fitted *r*^fit^, around the stable fixed-point. For *J* close to the saddle-node bifurcation (indicated by solid curve, gray circle) the fluctuations extend well beyond the unstable fixed-point (dashed curves), and thus the system can be pushed to the trivial attractor by a random fluctuation. For larger *J*, however, the basin of attraction is much larger than the population fluctuations (dashed-dotted curve), and thus lifetimes should become very long.

To relate these findings from the two-state Abeles-type model with Poisson input to the full recurrent SSAI, we perform the analogous analysis with some examples of the data we obtained from the systematic large-scale simulations discussed in Figures 3, 4.

**Figure 10:**
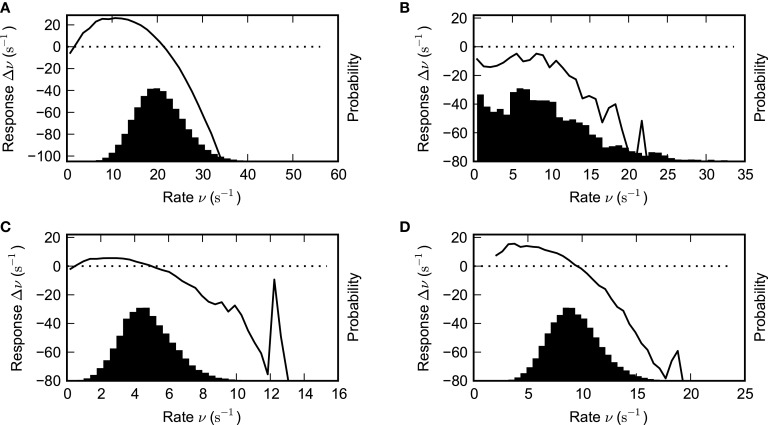
**Estimated network response function and population rate distribution**. The black solid lines depict the estimated network response functions, i.e., the local derivative of the firing rate function for various parameter combinations. It was estimated by computing the difference of input rate and output rate after a delay of δ*t* = 1.5 ms (see text for details). The histograms quantify the amount of time spent at a given rate. Parameter values: **(A)** *g* = 4.2, *J* = 1.1 mV **(B)** *g* = 4.2, *J* = 0.8 mV **(C)** *g* = 5.2, *J* = 1.8 mV **(D)** *g* = 4.8, *J* = 1.8 mV. Other parameters as in Figure 1.

Such estimated response functions Δν are shown in Figure 10. The intersections of the response function with the *x*-axis (dashed line in Figures 10A–D) again determine the fixed-points, while the slope at this points yields information about their stability: If the slope is positive, we expect the fixed-point to be unstable.

In the cases where the synapses are sufficiently strong to sustain persistent activity, we see that the distribution may be well approximated by a Gaussian centered at the upper fixed-point of the response function. This observation thus motivates the following simple stochastic model for the rate: We assume that the rate at any time is distributed normally with a mean given by the fixed-point of the response function. Both the response function and the width of the distribution are functions of the network and neuron parameters.

The probability to observe a given rate ν is thus,

(28) $$P_{g,J}(\nu )\propto e^{-\frac{{\left(\nu -\nu_{0}\right)}^{2}}{2\sigma^{2}}},$$

where ν_0_ = ν_0_(*g, J*) is the fixed-point of the response function, and σ = σ(*g, J*) is the width of the rate distribution.

From the observations of network response functions we can also see that there is indeed typically another (unstable) fixed-point λ close to the trivial fixed-point at zero. For the purpose of the stochastic rate model, we assume that if the rate fluctuates to a value less than λ, the network activity will move toward the trivial fixed-point at zero rate and cease.

From the probability distribution above, we can calculate the probability for the rate to be below λ, i.e.,

(29) $$P(\nu <\lambda )\;\propto \int_{-\infty }^{\lambda }e^{-\frac{{\left(\nu -\nu_{0}\right)}^{2}}{2\sigma^{2}}}d\nu =\frac{1}{2}\left(1-\text{erf}\left[\frac{\lambda -\nu_{0}}{\sqrt{2}\sigma }\right]\right).$$

We conclude that the lifetime for the self-sustained network activity will be inversely proportional to the probability for the network activity to cease,

(30) $$T(g,J)=\frac{\tau_{0}}{P(\nu <\lambda )},$$

where τ_0_ is a constant (see also El Boustani and Destexhe, 2009). Thus, the lifetime is determined by a trade-off between the magnitude σ of the population-rate fluctuations and the size ν_0_ − λ of the basin of attraction of the non-trivial rate fixed point.

#### 3.4.1. Performance of the stochastic model in predicting SSAI lifetime

We validate the stochastic model approach Equations (29), (30) by estimating the values for ν_0_, λ and σ, as well as the lifetimes *T*, from network simulations for a range of values for the parameters *g* and *J* and fitting the parameter τ_0_ using Equation (30).

**Figure 11:**
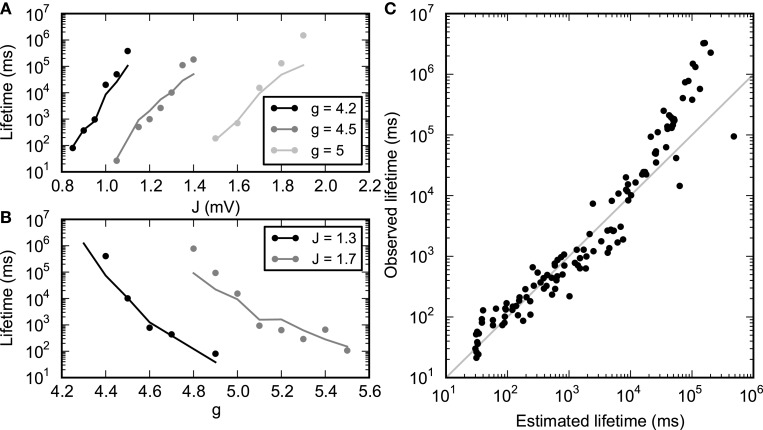
**Lifetime of SSAI-states**. Lifetime of SSAI-states estimated from measured parameters (solid lines) using Equation (30) with τ_0_ = 15 ms and observed directly (dots). **(A)** Lifetime for selected values of inhibition level *g*. **(B)** Lifetime for selected values of synapse strength *J*. **(C)** Observed vs. estimated lifetimes for several simulations with different parameters (*g, J*). Other parameters as in Figure 1.

The values for the parameters ν_0_ and λ as a function of *g* and *J* were found by inspection of the response functions obtained by the method described in the previous section. The measured response curves, being averages over the full simulation, are noisier and less smooth when the lifetime of persistent activity is short. For longer lifetimes, the points ν_0_ and λ were found using an automated approach, using linear interpolation between the points in the measured response curve. For the more noisy curves, the points were estimated manually by inspecting the response curves. The value for σ was the standard deviation of the instantaneous population rate observed during the simulation. Figure 11 shows the estimated and measured lifetimes for a range of values of *g* and *J*, revealing a good agreement.

We note that a saddle-node bifurcation as predicted from the Abeles-type two-state model Equation (19) is also predicted from the diffusion-approximation (Brunel, 2000 and Equation S1 in the Supplementary Material) for strong enough coupling strength *J*. The respective saddle-node bifurcation lines for Equation (19) and the diffusion-approximation are depicted for reference as white lines in Figure 3A. From these equations we can thus also derive at least qualitative predictions for the lifetime without having to estimate parameters from simulations. The resulting plots are presented in the Supplementary Material Section 1.

## 4. Discussion

### 4.1. Self-sustained activity in networks of LIF neurons with current-based synapses

Local cortical circuits can sustain elevated levels of activity after removal of the original stimulus or in total absence of external drive. Moreover, this ongoing activity is often characterized by highly fluctuating individual firing rates. In contrast to previous beliefs (see e.g., Kumar et al., 2008; El Boustani and Destexhe, 2009), here we demonstrate that balanced random networks with strong current-based synapses can actually combine both features: the sustained asynchronous activation of groups of neurons in the absence of external drive together with the highly irregular spiking of individual cells. We call this state self-sustained asynchronous-irregular, or SSAI.

We analyzed and identified simple mechanistic explanations for these activity features. The emergence of a stable attractor at non-zero rates is due to a saddle-node bifurcation: At sufficiently large synaptic efficacy, two fixed-points with finite rate exist in addition to the quiescent mode. These modes exist even when there is no external input to the network. The intermediate low-rate fixed-point is always unstable, while the fixed-point at higher rate can be long-lived with a lifetime rapidly increasing with synaptic efficacy.

Using a simple stochastic rate model, we have shown that the lifetime is determined by a trade-off between the size of the basin of attraction of the high-rate fixed-point and the intrinsic variance of the network activity in this state. The stochastic model explains the lifetime over a wide range of network parameters.

### 4.2. Origin of irregular SSA in a two-state Abeles-type model

The saddle-node bifurcation appears also in the simplified analytical models introduced by Siegert (1951), Griffith (1963) (see Supplementary Material Section 1) and Abeles (1982). Here we showed in particular, how a simple two-state Abeles-type model can be translated to the specific case of leaky integrate-and-fire (LIF) neurons with subthreshold linear dynamics. We find that in the SSAI state most of the time individual neurons will be strongly hyperpolarized and far below threshold, but at times a large depolarizing input transient will occur that will drive neurons repetitively across threshold in a short time, leading in effect to highly irregular firing.

We note that quantitatively the two-state model yields good agreement with the observed SSAI-states, if the amplitude of the free membrane potential fluctuations is large, and their mean and variance are known. The latter can be measured in simulations, but in practical terms they are hard to assess. For other cases, such as the Poisson-driven LIF-ensemble shown in Figures 5C,D, 6A, the rate prediction was too low, even though in this case, mean and variance of *V*_free_ are directly obtained from the firing rate. Part of the reason for the too low firing rate is the smaller amplitude of fluctuations. Another reason is that the self-consistent solution obtained from the Poisson-input scenario implicitly assumes that sampling spike trains from the output of neurons in turn yields a Poisson process again. This is clearly not the case, since every individual spike train will typically be non-Poissonian with a CV higher than unity, as we discussed in Section 3.2.2. Neurons sampling from the Poisson-driven pool in Figure 6A already have increased rate, CV and σ[*V*_free_], see Figure 6B. So a more quantitative self-consistent two-state Abeles model would have to incorporate a better spike train model, capturing more of the true “binary” statistics we observed here.

Still, our model nicely shows that high variability of the spiking activity of individual neurons, pronounced population fluctuations, and stable persistent activity can go together well (see also Druckmann and Chklovskii, 2012 for a related discussion), unlike previously thought (Kumar et al., 2008), and be realized by simple networks of integrate-and-fire neurons. Indeed, both during up-states (e.g., Shu et al., 2003, and persistent mnemonic states in prefrontal cortex e.g., Compte et al., 2003a), CVs of ISIs considerably larger than unity are common. We expect the effects reported here also in spiking network models of working memory that contain a stable low-rate attractor (which is not present in the simple network analyzed here), if they have a finite amount of comparably strong synapses. Such a network mechanism for the generation of fluctuating individual firing rates as presented in this paper could avoid the necessity to introduce additional noise sources or cellular bistability to obtain this effect (see e.g., Renart et al., 2003; Compte, 2006).

### 4.3. Highly hyperpolarized membrane potentials as side effect of membrane potentials without lower bound

Broad membrane potential distributions as observed here are not very physiological and not possible for neurons with conductance-based synapses (Kuhn et al., 2004), because of the limiting effect of the respective reversal potentials for NMDA or AMPA in the case of excitation, and GABA for inhibition.

Yet, also in networks of leaky integrate-and-fire neurons with conductance-based synapses self-sustained activity states occur for broad parameter ranges of excitatory and inhibitory conductances (Vogels and Abbott, 2005; Kumar et al., 2008; El Boustani and Destexhe, 2009). The self-sustained activity state analyzed there usually requires large networks sizes and low population rate fluctuations to be stable (Kumar et al., 2008; El Boustani and Destexhe, 2009) and is much more sensitive to subthreshold perturbations than the networks investigated here. The coefficient of variation (CV) of the inter-spike intervals can be larger than unity, indicating that spiking is more irregular than Poisson (Kumar et al., 2008; Teramae et al., 2012; Ikegaya et al., 2013), yet observed CVs in the networks studied in these papers are typically smaller than those we report here for neurons with current-based synapses (see however, Vogels and Abbott, 2005; El Boustani and Destexhe, 2009, that report parameter regimes with CVs in the range of two to three).

In the Supplementary Material Section 6, we demonstrate cases of self-sustained activity in comparably small networks of neurons with conductance-based synapses where CVs of the inter-spike intervals are considerably larger than unity, and the membrane potential distributions are typically also comparably broad. Similar arguments as presented here for current-based synapses thus explain this higher variability and show that large network size is not a requirement for SSAI, as suggested by previous work (Kumar et al., 2008; El Boustani and Destexhe, 2009).

We moreover note that clamping the membrane potential of LIF neuron with current-based synapses at a minimal value to avoid unbiological hyperpolarization leads to a shift of the saddle-node bifurcation line to smaller *J*-values. This is due to the fact that the membrane potential distribution is shifted closer to firing threshold, see Supplementary Material Section 4.

Asynchronous, highly irregular self-sustained activity, even in comparably small, yet strongly coupled networks, does thus not crucially depend on the synapse model, nor on extremely large subthreshold membrane potential fluctuations, but it is mainly a consequence of the large input fluctuations generated by the highly variable neuronal activities and the strong synaptic weights.

### 4.4. Few strong weights sufficient for emergence of SSAI

We emphasize that a comparably small fraction of strong weights suffices to permit self-sustained activity (see Teramae et al., 2012; Gewaltig, 2013; Ikegaya et al., 2013, and Supplementary Material Section 5), and such weights are not unbiological. Indeed, recent experiments consistently showed that the presence of strong synapses is not uncommon in cortical and hippocampal networks, but rather the norm (Song et al., 2005; Lefort et al., 2009; Avermann et al., 2012; Ikegaya et al., 2013). Weight distributions follow a lognormal distribution that is characterized by a high probability for low weights, but a heavy tail probability for very strong synapses, up to several millivolts. These weight distributions are usually characterized by high variances.

The reduced Abeles-type model already shows that the critical average coupling strength for the saddle-node bifurcation decreases, if the variance of the weight-distribution increases. For the extreme case of mostly very weak synapses and few very strong synapses, the reduced model predicts SSAI to occur for small average coupling strength on the order of *J* ~ 0.1 mV. This observation explains the related finding by Ikegaya et al. (2013) that deletion of the strongest weights quickly leads to failure of SSAI.

Song et al. (2005) moreover showed that strong synapses preferentially occur organized non-randomly in structural reciprocal motifs. It is thus an interesting question in this context, whether several strongly connected cell-assemblies of current-based leaky integrate-and-fire neurons in a sea of weak synapses can be activated selectively as suggested, e.g., in Brunel (2003), without activating other local attractors or the whole network, and if such activation is stable to “distractor” activation from other parts of the network, as would be required, e.g., in working memory.

### 4.5. Effects of strong synapses in complex random networks

The emergence of a self-sustained activity state is not the only intriguing dynamical effect caused by the presence of strong synapses. As pointed out in many studies, strong coupling in complex networks can lead to a breakdown of linearity and give rise to new collective phenomena, such as pattern formation, oscillations or traveling waves (see e.g., Amari, 1977; Ben-Yishai et al., 1995; Usher et al., 1995; Bressloff and Coombes, 1998, 2000; Roxin et al., 2005; Kriener et al., 2014).

The presence of strong synapses was shown to lead to spike-based aperiodic stochastic resonance, and thus reliable transmission of spike patterns, in an optimal self-sustained background regime in networks of conductance-based LIF neurons (Teramae et al., 2012). Moreover, strong synaptic weights in the same random network as discussed here will render the globally synchronous firing mode unstable to any finite perturbation, and thus stabilize the asynchronous-irregular state, even if all neurons receive statistically identical input of equal magnitude (Kriener, 2012).

Analogous to our observations, Ostojic (2014) in a recent paper observed how strong weights lead to highly irregular spiking with individually strongly fluctuating neuronal firing rates in the same networks analyzed here, but where neurons receive constant external drive. Similar observations of asynchronous and highly irregular states were made before for networks of rate neurons (Sommers et al., 1988), as well as spatially structured networks of spiking neurons that non-linearly amplify heterogeneous activity fluctuations (see e.g., Usher et al., 1994, 1995).

Ostojic, as well, explains the effects in random networks by the breakdown of the linear response approximation and the non-linear network amplification of heterogeneous perturbations (see detailed discussion in the Supplementary Material Section 2), and he identifies the emerging state as a qualitative different and new asynchronous-irregular state. He shows that in this state average firing rates characteristically deviate to higher values as compared to the weakly-coupled balanced random network states analyzed by Brunel (2000). Most of all the heterogeneity of activity brings about interesting computational properties in classifying temporally fluctuating inputs (Ostojic, 2014).

The amplification by the recurrent network is also the reason that underlies the strengthening of irregularity and population fluctuations that we observe, e.g., in Figures 6A–C, where we increase the effect of network feedback from initial Poisson-drive (zero-order feedback), over sampling from the resulting output (first-order feedback) to the full self-consistent SSAI (∞-order feedback). We showed in Section 3.2 that highly irregular spiking can already be observed in the uncoupled population of neurons fed with strongly weighted Poisson input, and even Gaussian white noise with high variance and strongly negative mean, in which case the firing rate does not deviate from that predicted by the diffusion-approximation (not shown). We note that although the breakdown of linear response theory with increasing coupling strength *J*, analyzed by Ostojic (2014), does not coincide with the emergence of the self-sustained activity state (see Supplementary Material Section 2), it does approximately overlap with the abrupt increase in firing rates to values ν_0_ ≥ 10 s^−1^ in Figure 3B, as well as of the CV to values ≥ 2.5 as shown in Figure 4A. We can thus conclude that we see the presence of this new qualitative state identified by Ostojic also in our simulations. This non-linear amplification effect might serve to stabilize SSAI by moving the population firing rate to higher values and thus farther away from the trivial fixed-point.

The existence of strong synapses in recurrent neuronal networks as observed in experiments thus leads to a plethora of interesting dynamical properties that just start to be explored, and analysis of how circuits can make use of their presence computationally is an important topic of future research.

## Author contributions

Birgit Kriener and Håkon Enger conceived and performed simulations, mathematical analysis, and data analysis. Birgit Kriener, Håkon Enger, Tom Tetzlaff, Hans E. Plesser, Marc-Oliver Gewaltig, and Gaute T. Einevoll wrote the manuscript.

### Conflict of interest statement

The authors declare that the research was conducted in the absence of any commercial or financial relationships that could be construed as a potential conflict of interest.

## Footnotes

^1^If we assume absolute refractoriness, i.e., the neuron loses the input during that period, the dynamics becomes biased toward higher rate because the neuron stays at *V*_res_ in the presence of the net-inhibitory input from the network. In the actual system that will be considered in Section 3.4 this is indeed the case, and the clamping at *V*_res_ during τ_ref_ is explicitly taken into account in the diffusion limit solution in Brunel (2000)
