# Supplementary material for: Dynamics of self-sustained asynchronous-irregular activity in random networks of spiking neurons with strong synapses
Source: Front Comput Neurosci. 2014 Oct 30;8:136. doi: 10.3389/fncom.2014.00136 (PMC4214205; doi:10.3389/fncom.2014.00136)
Supplement: Supplementary file 1 [file DataSheet1.PDF]

Frontiers in Computational Neuroscience

Research Article
15 August 2014

Supplementary Material: Dynamics of self-sustained asynchronous-irregular activity in random networks of spiking neurons with strong synapses

Birgit Kriener [1,2,*,†], Håkon Enger [2,3,†], Tom Tetzlaff [4], Hans Ekkehard Plesser [2], Marc-Oliver Gewaltig [5], and Gaute T. Einevoll [2,6]

[1] *Center for Learning and Memory, University of Texas at Austin, Austin, Texas, USA*
[2] *Department of Mathematical Sciences and Technology, Norwegian University of Life Sciences, Ås, Norway*
[3] *Kalkulo AS, Simula Research Laboratory, Fornebu, Norway*
[4] *Institute of Neuroscience and Medicine (INM-6), Computational and Systems Neuroscience & Institute for Advanced Simulation (IAS-6), Research Center Jülich, Jülich, Germany*
[5] *Blue Brain Project, École Polytechnique Fédérale de Lausanne, Lausanne, Switzerland*
[6] *Department of Physics, University of Oslo, Oslo, Norway*
[†] *These authors contributed equally*

Correspondence*:
Birgit Kriener
Center for Learning and Memory, University of Texas at Austin, Austin, Texas, USA, kriener@utexas.edu

1 COMPARISON OF DIFFERENT MODELS TO DERIVE THE SELF-CONSISTENT RATE AND SADDLE-NODE CURVE

1.1 GRIFFITH MODEL

One of the first to derive a firing rate model from first principles was Griffith (**Griffith**, 1963), and the basic idea is closely related to the Abeles-type model **Abeles** (1982). Griffith considered simple populations of N dynamically identical spiking neurons where the probability of emitting a spike within a given fixed time bin Δt between two iteration steps is given by a parameter $p \in [0, 1]$. p is thus the firing probability, while the finite interval Δt serves as the memory time window of the neuron in this time-discrete dynamics. In a recurrent network the probability to emit a spike is proportional to the probability $f_{\Delta t}$ to receive sufficient suprathreshold input. $f_{\Delta t}$ is a function of p, and a self-consistent solution must fullfil

$$f_{\Delta t}(p) = p\,. \tag{1}$$

As shown by **Griffith** (1963), this equation has indeed non-trivial solutions, i.e., self-sustained activity, when the threshold is low enough, corresponding to high synaptic efficacy.

1.2 SIEGERT MODEL

The full recurrent dynamics of balanced random networks of LIF neurons with current-based synapses was extensively studied in the seminal papers by **Amit and Brunel** (1997) and **Brunel** (2000), who demonstrated how neuronal firing rate and spiking irregularity can be quantitatively derived by self-consistently solving a Fokker-Planck equation. The solution for the self-consistent rate was first derived by **Siegert** (1951) as the solution of the mean first-passage time problem of a LIF neuron receiving Gaussian white noise, and it is given by

$$\nu_0^{-1} = \tau_{\mathrm{ref}} + \tau_{\mathrm{m}}\sqrt{\pi}\int_{\frac{V_{\mathrm{res}}-\mu_0}{\sigma_0}}^{\frac{V_{\mathrm{thr}}-\mu_0}{\sigma_0}} \exp[x^2]\,(1+\mathrm{erf}[x])\,dx\,, \tag{S1}$$

where $\mu_0 = \mu(\nu_0)$ and $\sigma_0 = \sigma(\nu_0)$ are the self-consistent mean and standard deviation of the input current, τ_{ref} is the refractory time and erf[.] denotes the error function.

Two core assumptions underlying this solution are that the synaptic coupling strength is weak and that spiking is Poissonian and uncorrelated. The network can assume various collective activity states that can be predicted from stability analysis of the stationary solution, such as asynchronous and irregular spiking (cf. Fig. 2A in the main manuscript), or fast global oscillations, only dependent on the synaptic coupling strength J, the relative inhibition g, and the strength of external input I_{ext} (**Brunel**, 2000).

1.3 RATE AND LIFETIME

To estimate the lifetime without running a network simulation to extract the rate model parameter, we need values for the fixed points ν_0 and λ and the distribution width σ. The "Siegert formula" (see **Siegert** (1951) and Eqn. (S1)) for the rate response function or the "Abeles" and "Griffith model" (**Abeles**, 1982; **Griffith**, 1963) can give estimates for ν_0 and λ, cf. Sec. 2.2 in the main manuscript. In Fig. 1 we compare the rate prediction from the Siegert formula to simulation results as a function of g and J. The figure also

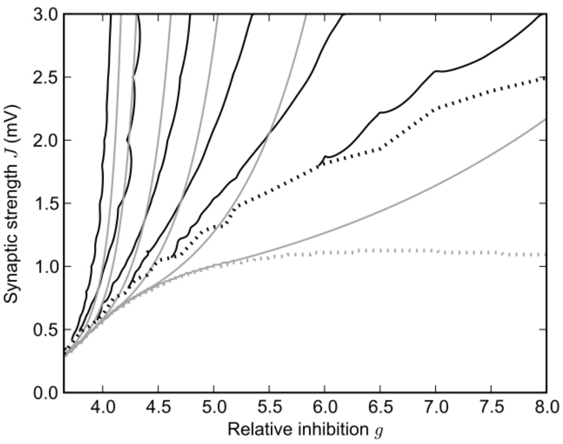

Figure 1. Self-consistent rate in simulations versus the Siegert prediction for networks with δ-type synapses. Comparison of rate (contour lines at $2, 5, 10, 20, 50$, and $100\,\mathrm{s}^{-1}$, right to left) and saddle-node curve (dotted lines) from the Siegert formula (Eqn. (16) in the main manuscript, gray) and observations in numerical experiments (black).

shows the curve in g-J-space where the second and third fixed point of the response function appear, i.e., the saddle point curve. We see that the Siegert formula is a good approximation to the observed rate when g is close to four, i.e., when inhibition and excitation are approximately balanced. The main discrepancy

between the Siegert formula and the observed results is the location of the saddle point for large values of g. The Siegert formula Eqn. (S1) predicts that the saddle point is at $J \approx 1\,\mathrm{mV}$, approximately independent of g for $g \gtrsim 5$. In the simulation results, the value of J at the saddle point is approximately linearly increasing with g.
Assuming that the LIF neuron may be approximated by the discrete Griffith model, on the other hand, we show the resulting saddle-point curve together with the corresponding curve for the Siegert formula and the estimate from the numerical experiments in Fig. 2. The simple Griffith-Abeles-type model prediction is slightly closer to what we observe in simulations for larger g.

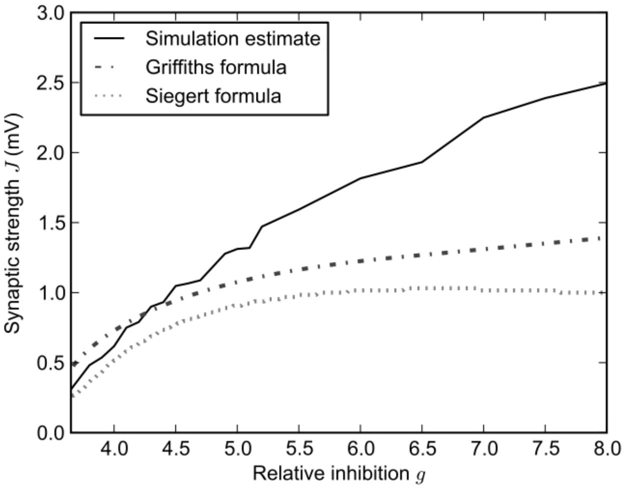

Figure 2. Saddle point curves comparison. Saddle point curves, separating the region in parameter space with three fixed points for the response function from the region with one, for Siegert formula, Griffith formula, and simulations (see legend).

2 CRITICAL COUPLING STRENGTH FOR THE BREAK-DOWN OF LINEARITY

In a recent paper **Ostojic** (2014) analyzes the effect of strong coupling in the same network architecture considered here, but for pulse-coupled LIF-oscillators, i.e., LIF neurons that are constantly driven by a suprathreshold direct current and coupled by instantaneous, i.e., δ-type synapses. For small coupling strength J and homogeneous asynchronous-irregular spiking, stability of this state to slow firing rate perturbations can be assessed by linear response theory (see, e.g., **Brunel** (2000); **De la Rocha et al.** (2007); **Tetzlaff et al.** (2012)) in which the dynamics of fluctuations $r(t)$ around the steady state ν_0 takes the linear form

$$\mathbf{r}(t) = ([-\mathbf{W}\mathbf{r} + \mathbf{x}] * h)(t) \tag{S2}$$

with the effective gains $W_{ij} = w(J)$, if j is excitatory, and $W_{ij} = w(-gJ)$ for inhibitory j, given by

$$w(J) = \frac{\partial \nu_0}{\partial \mu_0} \tau_m J + \frac{\partial \nu_0}{\partial \sigma_0} \frac{\tau_m}{2\sigma_0} J^2\,. \tag{S3}$$

Here, ν_0 is the Siegert equation given by Eqn. (S1), x is pairwise uncorrelated white noise (related to the white noise spectrum generated by the spiking of neurons) and $h(t)$ is the linear response kernel, see (**Tetzlaff et al.**, 2012) for a detailed derivation and discussion.

For increasing coupling strength J, this linear response approximation Eqn. (S2) breaks down, and at a critical strength J_{lin} the network becomes linearly unstable. This critical strength is derived by random

matrix theory, i.e., the J for which $\rho(J) = 1$, with

$$\rho(J) = w(J)\sqrt{N\epsilon\,(1-\epsilon)\,(\beta + \tilde{g}(J)^2(1-\beta))}\,, \tag{S4}$$

and

$$\tilde{g}(J) = -w(-gJ)/w(J)\,. \tag{S5}$$

W is a row-normalized random matrix with one right eigenvector proportional to $(1,\ldots,1)^\top$, and $(N-1)$ heterogeneous eigenvectors. Beyond J_lin these heterogeneous perturbations with positive eigenvalues are amplified by the ongoing noise in the network, leading to large variability of both individual firing rates, as well as rates across neurons (**Ostojic**, 2014).

The respective J_lin as a function of g and J for δ-type synapses is shown in Fig. 3 B in red. In the simulations in the main manuscript, however, we used synapses with α-type PSCs. For the short synaptic time constant used there ($\tau_\text{syn} = 0.5$ ms) we expect the all-over picture with respect to the g-J-plane in Figs. 3 and 4 in the main manuscript to look very similar for α and δ-type synapses.

For α-type synapses there is no stability theory available, yet. We can, however, make use of a mapping for exponential PSCs with small $\tau_\text{syn}/\tau_\text{m}$ derived by **Fourcaud and Brunel** (2002), i.e., for PSCs and PSPs, such that

$$\text{PSC}(t) = A(J_{ij})\,\text{exp}[-t/\tau_\text{syn}]\,H(t) \tag{S6}$$

$$\text{PSP}(t) = R_\text{m}A(J_{ij})\frac{\tau_\text{syn}}{\tau_\text{m}-\tau_\text{syn}}\left(\text{exp}[-t/\tau_\text{m}] - \text{exp}[-t/\tau_\text{syn}]\right)\,H(t)\,.$$

Here, $H(t)$ is the Heaviside step-function, $A(J_{ij})$ the current peak-amplitude of the PSC, and R_m is the membrane resistance. The Siegert equation in the small $\tau_\text{syn}/\tau_\text{m}$-limit becomes

$$\nu_0^{-1} = \tau_\text{ref} + \tau_\text{m}\sqrt{\pi}\int_{\frac{V_\text{res}-\mu_0}{\sigma_0}+\alpha\sqrt{\frac{\tau_\text{syn}}{\tau_\text{m}}}}^{\frac{V_\text{thr}-\mu_0}{\sigma_0}+\alpha\sqrt{\frac{\tau_\text{syn}}{\tau_\text{m}}}} \text{exp}[x^2]\,(1+\text{erf}[x])\,dx\,, \tag{S7}$$

with $\alpha = |\zeta(1/2)|/\sqrt{2} \approx 1.033$ and Riemann zeta-function $\zeta(x)$ (**Fourcaud and Brunel**, 2002).

We can now numerically fit $A(J_{ij})$ and τ_syn of the exponential synapse to match the PSP(t) of the α-synapse (see Fig. 3 A and Eqn. (5) in the main manuscript) and evaluate the derivative of Eqn. (S7) in Eqn. (S3) to obtain the theoretical expectation for the breakdown of linear response theory. The result is shown in Fig. 3 B (gray) in comparison to the δ-synapse prediction (red). Indeed, they give similar quantitative predictions.

We note that neither critical line for linear breakdown $J_\text{lin}(g, J)$ coincides with the saddle-node bifurcation line, cf. Fig. 3 and Fig. 3A. They do, however, agree with the transition to very high CV in Fig. 4A in the main manuscript, as well as with the transition to firing rates $\nu_0 > 10/s$ in Fig. 3B. Thus, the nonlinear network amplification certainly plays a role in boosting irregularity and increasing the firing rate.

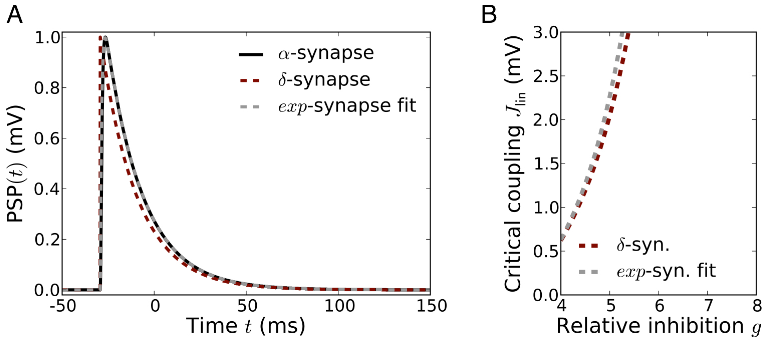

Figure 3. Critical coupling strength J_{lin} for breakdown of linear response theory. A The PSP(t) for δ-type synapses (red), α-type synapses (black, cf. Eqn. (4) in the manuscript) and exponential synapses (gray, cf. Eqn. (S6)) for the parameters used throughout in the simulations. Here, the exponential synapse parameters $J^{exp} = 1.14\,J_{ij}$ and $\tau_{\text{syn}}^{exp} = 1.6\,\tau_{\text{syn}}$ were numerically fit to match the time course of the α-type synapse. **B** The critical coupling strength J_{lin} at which linear response theory breaks down in the SSAI-state as a function of g and J, assuming δ-type or exponential synapses matched to the time-course of the α-type synapses used in the main manuscript. Other parameters: $\tau_{\text{syn}}^{\alpha} = 0.5$, $N = 125\,000$, $\epsilon = 0.01$, $V_{\text{thr}} = 20\,\text{mV}$, $V_{\text{res}} = 0\,\text{mV}$, $R_{\text{m}} = 80\,\text{M}\Omega$, $\tau_{\text{m}} = 20\,\text{ms}$, $\tau_{\text{ref}} = 2\,\text{ms}$, $d = 1.5\,\text{ms}$.

3 ONE SPIKE-PERTURBATION SUFFICES TO ACTIVATE THE SSAI-STATE IN THE $J \rightarrow \theta$ – LIMIT

The Abeles-model for the network-parameters considered here predicts that for large J-values the unstable intermediate fixed point moves towards the zero-rate fixed point, see main manuscript, Sec. 3.2.3, Fig.7. Indeed, the smallest conceivable perturbation of the quiescent state in a spiking network, i.e., the emission of one spike, can successfully trigger activation of the high-rate fixed point, if J is of the order of distance between resting potential and threshold θ, implying that one excitatory spike can elicit a spike in a post-synaptic neuron. This is shown in Fig. 4.

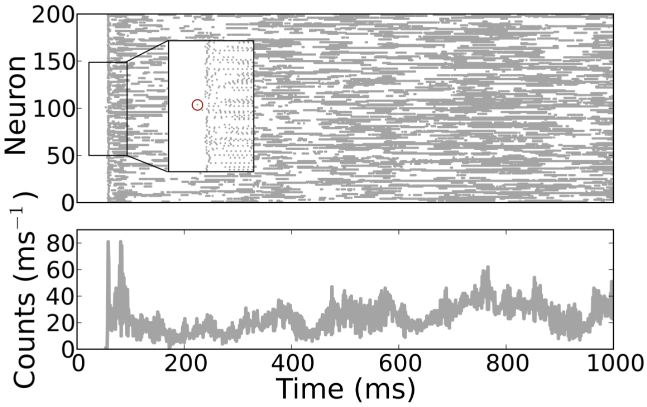

Figure 4. One excitatory spike triggers SSAI-state. Demonstration that a single excitatory spike, delivered at 50 ms to neuron 100, suffices to trigger a network to enter a stable (longer than simulation time of 40 s lifetime) SSAI-state. Shown are 200 randomly chosen neurons in a network of $N = 5000$ neurons, $N_{\text{I}} = N_{\text{E}}/4$, with connection density $\epsilon = 0.1$, $J = \theta = 20\,\text{mV}$, $g = 4.2$. All other parameters as specified in table 5.2 in the main manuscript.

4 SELF-SUSTAINED ACTIVITY STATES IN RANDOM NETWORKS OF LIF NEURONS WITH LOWER MEMBRANE POTENTIAL BOUND

Because in the LIF neuron model with current-based synapses used throughout the main manuscript there is no lower bound for the membrane potential $V(t)$ it tends to go to unbiological, extremely hyperpolarized values in the SSAI state. This can be alleviated by introducing a reflecting lower bound at a reasonable value such as $V_{\text{min}} = V_{\text{thr}} - 40\,\text{mV}$. This does not impede the self-sustained activity state, but even facilitates it, because the bulk membrane potential distribution will stay closer to threshold. Fig. 5 demonstrates stable SSAI for the identical network setup as in Figs. 5 and 6 in the main manuscript, but for $J = 2.0\,\text{mV}$ and the existence of a lower bound of the LIF neurons at $V_{\text{min}} = -20\,\text{mV}$.

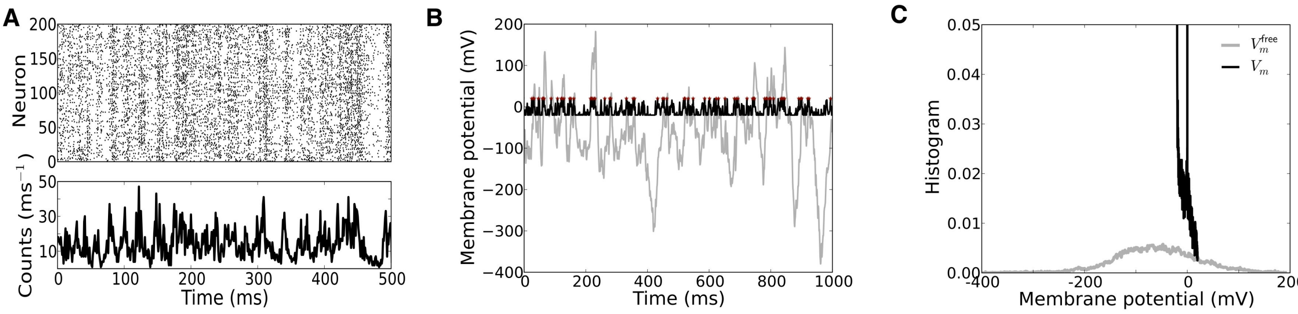

Figure 5. SSAI in networks of LIF neurons with reflecting lower membrane potential bound. A shows the spikes (upper panel) of 200 randomly chosen neurons and the population activity (lower panel) of a network of $N_{\text{E}} = 4000$ excitatory and $N_{\text{I}} = 1000$ LIF neurons with a connection density of $\epsilon = 0.1$ for a period of 500 ms. The network stayed active after an initial stimulation period for the total duration of the simulation (10 s). **B** shows the membrane potential $V(t)$ (black), free membrane potential $V_{\text{free}}(t)$ (gray) and spikes (red asterisks) of one randomly chosen neuron from that network. **C** depicts the respective histogram of $V(t)$ (black) and $V_{\text{free}}(t)$ (gray). The free membrane potential is the input current filtered by the membrane and can thus go to very hyper- and depolarized values, while the actual membrane potential is limited by the threshold $V_{\text{thr}} = 20\,\text{mV}$ above, and by the reflecting boundary $V_{\text{min}} = -20\,\text{mV}$ below. The peaks in the histogram of $V(t)$ correspond to V_{min} and the reset potential $V_{\text{res}} = 0\,\text{mV}$. Other parameters: $J = 2.0\,\text{mV}$, $g = 4.2$, $\tau_{\text{m}} = 20\,\text{ms}$. Synaptic PSCs were α-functions with $\tau_{\text{syn}} = 0.5\,\text{ms}$, see main manuscript.

5 SELF-SUSTAINED ACTIVITY STATES IN RANDOM NETWORKS OF LIF NEURONS WITH LOGNORMALLY DISTRIBUTED SYNAPTIC WEIGHTS

In Sec. 3.3 of the main manuscript we discussed the effect of a finite variance of the distribution of synaptic weights and how it can lead to a reduction of the mean critical coupling strength J_c for the saddle-node bifurcation to occur. In particular we discussed the effect of lognormally distributed weights that are characterized by a skewed distribution with a high density of weak weights and a heavy tail of strong weights, see Fig. 6 D.

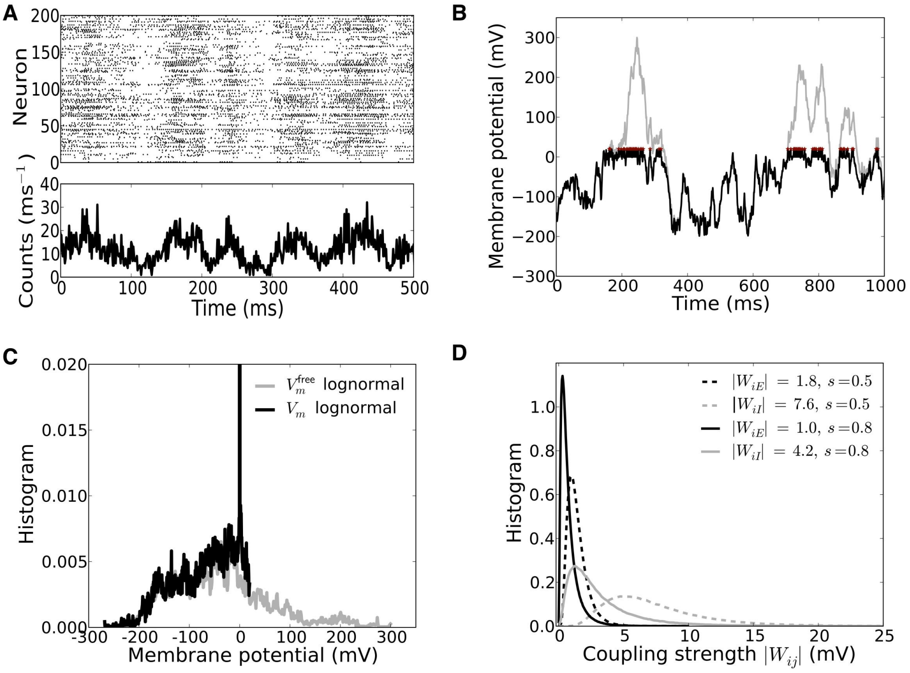

Figure 6. SSAI dynamics in networks with lognormal weight distribution. A shows the spikes (upper panel) of 200 randomly chosen neurons and the population activity (lower panel) of a network of $N_{\mathrm{E}} = 4000$ excitatory and $N_{\mathrm{I}} = 1000$ LIF neurons with a connection density of $\epsilon = 0.1$ for a period of 500 ms. The network stayed active after an initial stimulation period for the total duration of the simulation (10 s). **B** shows the membrane potential V_{m} (black), free membrane potential V_{free} (gray) and spikes (red asterisks) of one randomly chosen neuron from that network. **C** depicts the respective histogram of $V(t)$ (black) and $V_{\mathrm{free}}(t)$ (gray). **D** Histograms of coupling weights from two successful SSAI networks with weights drawn from a lognormal distribution. The solid lines depict the absolute value of the excitatory (gray) and inhibitory weights for $\mathrm{E}[|W_{iE}|] = J = 1.0\,\mathrm{mV}$ and $\mathrm{E}[|W_{iI}|] = gJ$, with $g = 4.2$. The parameter s was fixed to 0.8. The dashed lines show the same for $s = 0.5$ and $\mathrm{E}[|W_{iE}|] = J = 1.8\,\mathrm{mV}$, $\mathrm{E}[|W_{iI}|] = 4.2J$. Other parameters: $V_{\mathrm{thr}} = 20\,\mathrm{mV}$, $V_{\mathrm{res}} = 0\,\mathrm{mV}$, $\tau_{\mathrm{m}} = 20\,\mathrm{ms}$. Synaptic PSCs were α-functions with $\tau_{\mathrm{syn}} = 0.5\,\mathrm{ms}$, see main manuscript.

Here, we exemplarily show an example of random networks of LIF networks that can sustain activity in absence of external drive, cf. Fig. 6. All parameters are the same as in Figs. 5 and 6 of the main manuscript, however the mean absolute coupling strength J_c at the saddle-node bifurcation is lower. In Fig. 6 A-C for example, stable sustained activation is observed for $J = 1.8\,\mathrm{mV}$ for a network of size $N = 5000$, connection density $\epsilon = 0.1$ and relative inhibition $g = 4.2$.

The critical weight J_c mainly depends on the width of the distribution that in turn depends on the two parameters m and s of the lognormal distribution Log-$\mathcal{N}(m, s)$. The mean of the distribution is given by $\mathrm{E}[W_{ij}] = e^{m+s^2/2}$ and its median by e^m.

For fixed $\mathrm{E}[W_{ij}] = W$ we have $\mathrm{Log}[W] = m - s^2$, and hence the larger s is, the smaller m and thus the median will be. This means that for large s more weights will be small, while few will be very large.

Fig. 6 D shows two weight distributions that support SSAI. Both have smaller average weight amplitude, $\mathrm{E}[|W_{iE}|] = 1.0$ and $1.8\,\mathrm{mV}$ for excitation, and $\mathrm{E}[|W_{iI}|] = 4.2$ and $7.56\,\mathrm{mV}$ for inhibition, respectively, than that used in Figs. 5 and 6 of the main manuscript. Though there is a larger variability in survival rate of the SSAI for given $\mathrm{E}[W_{ij}]$ across realizations of coupling matrices in the case of a lognormal weight distribution, the general trend agrees very well with the discussion in Sec. 3.3 in the main manuscript: the larger s is, the lower the critical J_c for emergence of the second stable rate attractor will be, i.e., few strong weights in a sea of weak ones may suffice to keep activity going, see also **Ikegaya et al.** (2013).

6 SELF-SUSTAINED ACTIVITY STATES IN BALANCED RANDOM NETWORKS OF LIF NEURONS WITH CONDUCTANCE-BASED SYNAPSES

The subthreshold dynamics $V(t)$ of a leaky integrate-and-fire neuron with conductance-based synapses (coba LIF) is given by (see, e.g., **Kuhn et al.** (2004); **El Boustani and Destexhe** (2009) for a comprehensive overview)

$$C_\mathrm{m}\frac{d}{dt}V(t) = g_\mathrm{leak}(V_\mathrm{rest} - V(t)) + g_\mathrm{E}(t)(E_\mathrm{rev} - V(t)) + g_\mathrm{I}(t)(I_\mathrm{rev} - V(t)) \qquad \text{(S8)}$$

$$\frac{d}{dt}g_\mathrm{E/I}(t) = -\frac{g_\mathrm{E/I}(t)}{\tau_\mathrm{synE/I}} + \Delta g_\mathrm{E/I}\, S_\mathrm{E/I}(t-d)$$

where we assumed exponentially decaying dynamics $g_\mathrm{E/I}(t)$ with time constants $\tau_\mathrm{synE/I}$ for excitatory (E) and inhibitory (I) conductances, with instantaneous jumps by $\Delta g_\mathrm{E/I}$ whenever there is an incoming spike. $S(t)$ denotes these incoming spike trains, d is a transmission delay, C_m is the membrane capacity, V_rest the resting potential, while E_rev and I_rev denote the excitatory and inhibitory reversal potential, respectively. Whenever $V(t)$ hits a threshold value V_thr, it is instantaneously reset to $V(t^+) = V_\mathrm{rest}$, a spike is emitted, and the membrane potential is clamped at V_rest for a refractory period τ_ref. Examples of the resulting spike, membrane potential and population dynamics in the self-sustained activity state in random networks of excitatory and inhibitory coba LIF neurons are shown in Fig. 7.

ACKNOWLEDGMENTS

We gratefully acknowledge funding by the eScience program of the Research Council of Norway under grant 178892/V30 (eNeuro), the Helmholtz Alliance on Systems Biology, the Helmholtz Association in the Portfolio theme “Supercomputing and Modeling for the Human Brain”, the Jülich-Aachen Research Alliance (JARA), and EU Grant 269921 (BrainScaleS). All network simulations were carried out with NEST (http://www.nest-initiative.org, **Gewaltig and Diesmann** (2007)) using NOTUR computing resources.

REFERENCES

Abeles, M. (1982), Role of cortical neuron: integrator or coincidence detector?, *Israel Journal of Medical Science*, 18, 83–92

Amit, D. J. and Brunel, N. (1997), Dynamics of a recurrent network of spiking neurons before and following learning, *Network: Comput. Neural Syst.*, 8, 373–404

Brunel, N. (2000), Dynamics of sparsely connected networks of excitatory and inhibitory spiking neurons, *Journal of Computational Neuroscience*, 8, 3, 183–208

De la Rocha, J., Doiron, B., Shea-Brown, E., Kresimir, J., and Reyes, A. (2007), Correlation between neural spike trains increases with firing rate, *Nature*, 448, 16, 802–807

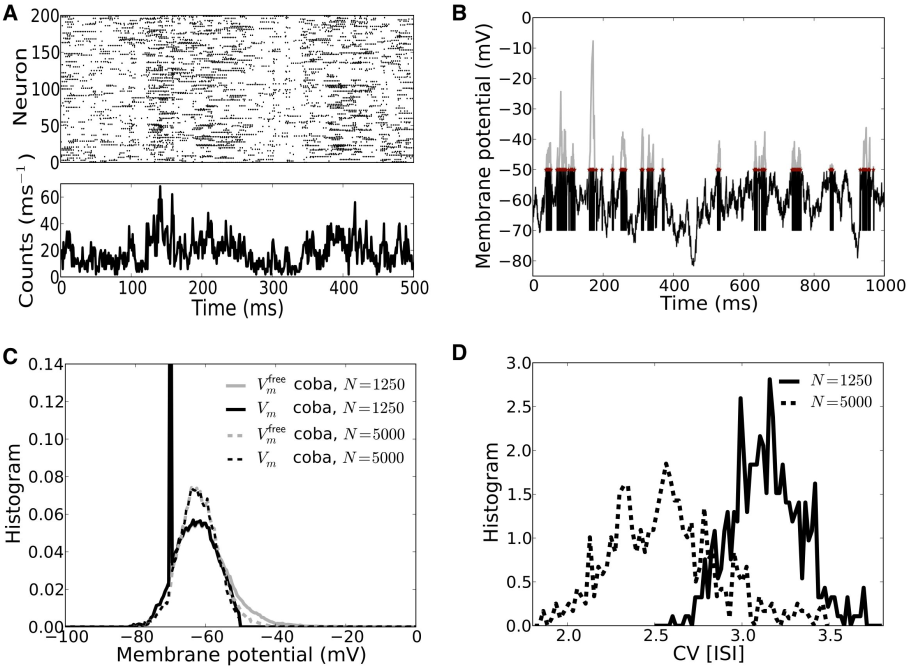

Figure 7. Highly irregular activity in random networks of leaky integrate-and-fire neurons with conductance-based synapses. Asynchronous self-sustained activity with highly irregular spiking and strongly fluctuating population rate can also be observed in networks of leaky integrate-and-fire neurons with conductance-based synapses (coba LIF, see Eqns. S8). **A** shows the spikes (upper panel) of 200 randomly chosen neurons and the population activity (lower panel) of a network of $N_{\mathrm{E}} = 4000$ excitatory and $N_{\mathrm{I}} = 1000$ coba LIF neurons with a connection density of $\epsilon = 0.1$ for a period of 500 ms. The network stayed active after an initial stimulation period for the total duration of the simulation (10 s). **B** shows the membrane potential $V(t)$ (black), free membrane potential $V_{\mathrm{free}}(t)$ (gray) and spikes (red asterisks) of one randomly chosen neuron from that network. **C** depicts the respective histogram of V_{m} and $V_{\mathrm{m}}^{\mathrm{free}}$ (solid lines), and of a neuron from a network of $N_{\mathrm{E}} = 1000$ excitatory and $N_{\mathrm{I}} = 250$ coba LIF neurons with same connection probability ϵ. For coba synapses the membrane potential distribution is limited by the respective reversal potentials E_{rev} and I_{rev} of excitation and inhibition (here $E_{\mathrm{rev}} = 0\,\mathrm{mV}$, $I_{\mathrm{rev}} = -90\,\mathrm{mV}$). Still we see that the free membrane potential shows comparably large excursion beyond the firing threshold V_{thr} (here at $-50\,\mathrm{mV}$), explaining the irregularity of spiking. This irregularity is quantified by the coefficient of variation (CV) of the interspike-intervals (ISI). If the CV is larger than (smaller than) unity, spiking is more (less) irregular as a Poisson process of the same rate. The distributions of CVs in the two shown examples are depicted in **D**. The mean CV is clearly larger than one. Other parameters: $\Delta g_{\mathrm{E}} = 7\,\mathrm{nS}$, $\Delta g_{\mathrm{I}} = 24.5\,\mathrm{nS}$, $g_{\mathrm{leak}} = 10\,\mathrm{nS}$, and $V_{\mathrm{rest}} - 70\,\mathrm{mV}$. Synaptic time constants $\tau_{\mathrm{synE}} = \tau_{\mathrm{synI}} = 5\,\mathrm{ms}$, delay $d = 2\,\mathrm{ms}$, and total refractory time $\tau_{\mathrm{ref}} = 2\,\mathrm{ms}$. The membrane capacitance was $C_{\mathrm{m}} = 200\,\mathrm{pF}$.

El Boustani, S. and Destexhe, A. (2009), A master equation formalism for macroscopicmodeling of asynchronous irregular activity states, *Neural Computation*, 21, 46–100

Fourcaud, N. and Brunel, N. (2002), Dynamics of the firing probability of noisy integrate-and-fire neurons, *Neural Computation*, 14, 2057–2110

Gewaltig, M.-O. and Diesmann, M. (2007), NEST (NEural Simulation Tool), *Scholarpedia*, 2, 4, 1430

Griffith, J. S. (1963), On the stability of brain-like structures, *Biophysical Journal*, 3, 299–308

Ikegaya, Y., Sasaki, T., Ishikawa, D., Honma, N., Tao, K., Takahashi, N., et al. (2013), Interpyramid spike transmission stabilizes the sparseness of recurrent network activity, *Cerebral Cortex*, 23, 293–304

Kuhn, A., Aertsen, A., and Rotter, S. (2004), Neuronal integration of synaptic input in the fluctuation-driven regime, *Journal of Neuroscience*, 24, 10, 2345–2356

Ostojic, S. (2014), Two types of asynchronous activity in networks of excitatory and inhibitory spiking neurons, *Nature Neuroscience*, 17, 594–600

Siegert, A. J. (1951), On the first passage time probability problem, *Physical Review*, 81, 4, 617–623

Tetzlaff, T., Helias, M., Einevoll, G., and Diesmann, M. (2012), Decorrelation of neural-network activity by inhibitory feedback, *PLoS Computational Biology*, 8, 8, e1002596
